# *Trichoderma* Applications on Strawberry Plants Modulate the Physiological Processes Positively Affecting Fruit Production and Quality

**DOI:** 10.3389/fmicb.2020.01364

**Published:** 2020-07-03

**Authors:** Nadia Lombardi, Simonetta Caira, Antonio Dario Troise, Andrea Scaloni, Paola Vitaglione, Francesco Vinale, Roberta Marra, Anna Maria Salzano, Matteo Lorito, Sheridan Lois Woo

**Affiliations:** ^1^Department of Agricultural Sciences, University of Naples Federico II, Naples, Italy; ^2^Proteomics and Mass Spectrometry Laboratory, ISPAAM, National Research Council, Naples, Italy; ^3^Department of Veterinary Medicine and Animal Productions, University of Naples Federico II, Naples, Italy; ^4^Institute for Sustainable Plant Protection, National Research Council, Portici, Italy; ^5^Task Force on Microbiome Studies, University of Naples Federico II, Naples, Italy; ^6^Department of Pharmacy, University of Naples Federico II, Naples, Italy

**Keywords:** *Fragaria x ananassa*, *Trichoderma*, proteomics, antioxidant, phenolics, anthocyanins

## Abstract

Many *Trichoderma* spp. are successful plant beneficial microbial inoculants due to their ability to act as biocontrol agents with direct antagonistic activities to phytopathogens, and as biostimulants capable of promoting plant growth. This work investigated the effects of treatments with three selected *Trichoderma* strains (T22, TH1, and GV41) to strawberry plants on the productivity, metabolites and proteome of the formed fruits. *Trichoderma* applications stimulated plant growth, increased strawberry fruit yield, and favored selective accumulation of anthocyanins and other antioxidants in red ripened fruits. Proteomic analysis of fruits harvested from the plants previously treated with *Trichoderma* demonstrated that the microbial inoculants highly affected the representation of proteins associated with responses to stress/external stimuli, nutrient uptake, protein metabolism, carbon/energy metabolism and secondary metabolism, also providing a possible explanation to the presence of specific metabolites in fruits. Bioinformatic analysis of these differential proteins revealed a central network of interacting molecular species, providing a rationale to the concomitant modulation of different plant physiological processes following the microbial inoculation. These findings indicated that the application of *Trichoderma*-based products exerts a positive impact on strawberry, integrating well with previous observations on the molecular mechanisms activated in roots and leaves of other tested plant species, demonstrating that the efficacy of using a biological approach with beneficial microbes on the maturing plant is also able to transfer advantages to the developing fruits.

## Introduction

For centuries, the fruits of strawberry (*Fragaria x ananassa* Duch.) have been consumed, appreciated for their taste and nutritional properties. Their cultivation has augmented in recent years, with productivity increasing to more than 9 million tons worldwide in 2017 (Food and Agriculture Organization of the United Nations, 2018)^1^. Human health benefits derived from eating strawberry fruits include heart protection, reduced blood pressure, as well as anticancer and anti-inflammatory activities (Liu et al., 2000; Joseph et al., 2014). These effects are related to the high content of phenolic compounds, vitamin C, anthocyanins, proanthocyanidins (cyanidin and pelargonidin derivatives) and other antioxidants, which contrast oxidative stress and retard cellular aging (Hanhineva et al., 2011; Giampieri et al., 2012; Joseph et al., 2014; Park et al., 2017). Anthocyanins represent the main flavonoid class in strawberry fruits that play an active role in the determination of the red pigmentation and in the evaluation of fruit ripeness (da Silva et al., 2007). Ripened strawberries contain high concentrations of pelargonidin glycosides, including pelargonidin 3-*O*-glucoside, pelargonidin 3-*O*-malonyl-glucoside, pelargonidin 3-*O*-rutinoside and cyanidin 3-*O*-glucoside, which may represent up to 95, 33, 7, and 6%, respectively, of the total anthocyanins (Aaby and Remberg, 2015). Previous studies reported that the biosynthesis of antioxidant metabolites (and the corresponding concentration in the fruit) is strongly influenced by the interaction of the plant genotype and cultivation practices, i.e., fertilization, with the growth environment (Anttonen et al., 2006; Buendia et al., 2010; Aaby et al., 2012). The metabolomic dynamics are also affected by the nutritional status of the plant, as noted with iron- and phosphorus- deficiencies in the rhizosphere, often compensated by the regulation of root exudates that influence nutrient bioavailability and uptake determining fruit quality (Valentinuzzi et al., 2015). Furthermore, metabolite composition of strawberry are also associated with the phenology of the plant, developmental stages of the fruit, vegetative structure and interactions with biotic and abiotic stress factors that correspond to plant responses to the pathogen/pest attack and to adverse factors in the environment (Hanhineva et al., 2011).

Recent findings reinforce the concept that some microbial biological control agents (BCAs) may have multiple beneficial effects on plants, that not only include disease control, but also the stimulation of plant growth, increased yield, enhanced bioavailability and uptake of nutrients, as well as improvement of crop quality (Pascale et al., 2017; Woo and Pepe, 2018; Marra et al., 2019). Numerous fungi belonging to the genus *Trichoderma* have been widely studied as BCAs for their antagonistic and plant biostimulant activities; they are present as active ingredients in more than 200 products marketed worldwide as biofungicides, biofertilizers, biostimulants, and soil probiotics for agriculture (Woo et al., 2014). Furthermore, many *Trichoderma* spp. applied as biofungicides and considered as alternatives to chemical phytosanitary products of synthesis, are proven efficient antagonists of many causal disease agents of strawberry such as *Botrytis* or *Colletotrichum* (Tronsmo and Dennis, 1977; Freeman et al., 2004; Porras et al., 2007).

Several *Trichoderma* strains are endophytes able to establish a complex molecular crosstalk network in interactions with other rhizosphere microorganisms and the plant, which improve plant feedback to different stresses, and the ability to improve crop development and productivity (Vinale et al., 2008; Hermosa et al., 2012; Lombardi et al., 2018).

Since the early observations of the plant growth promotion effects by *Trichoderma* treatments to the plant (Baker, 1988; Klefield and Chet, 1992; Ousley et al., 1994), investigations in this field of research have been steadily intensified in an attempt to understand the mechanisms involved. Root colonization by *Trichoderma* spp. was found to be associated with enhanced plant nutrient uptake as result of an improved efficient solubilization of macro- and micro-nutrients (Altomare et al., 1999; Yedidia et al., 2001; de Santiago et al., 2011) that modified the metabolism of several crops (Harman, 2004; Vinale et al., 2008, 2012; Hermosa et al., 2012). Modern technologies in the “omics” era permitted functional studies on the beneficial fungus (Lorito et al., 2010), the host plant (Harman et al., 2004a), as well as on the interactions between *Trichoderma*, plant and pathogens (Marra et al., 2006). Proteomic and transcriptomic approaches were used to characterize metabolic pathways and molecular processes underlying the plant response to treatments with *Trichoderma* preparations, specifically regarding the plant defense responses (Yedidia et al., 2000) and induced systemic resistance (Shoresh et al., 2010). The various studies that described the plant responses to these beneficial microbes were performed on root and leaf tissues obtained from diverse plant species, i.e., bean, maize, tomato, cucumber and grapevine. These plants were treated with *T. harzianum* or *T. virens* in order to evaluate the differential quantitative changes in proteins/genes related to specific signaling cascades and metabolic pathways involved in defense responses, redox stresses and carbon/energy metabolism (Marra et al., 2006; Segarra et al., 2007; Shoresh and Harman, 2008; Perazzolli et al., 2016; Manganiello et al., 2018; Nogueira-Lopez et al., 2018; De Palma et al., 2019). Although proteomics and metabolomics have found a large application in the characterization of the physiological changes occurring during development, ripening and post-harvest of diverse fruits (Guarino et al., 2007; D’Ambrosio et al., 2013; Molassiotis et al., 2013; Salzano et al., 2018, 2019), including strawberry (Bianco et al., 2009; Li et al., 2013, 2015), the objectives have been largely focused on the quality of the harvested products and the effects of conservation conditions.

The principle method to administer the biological products containing *Trichoderma* in agricultural production is by direct applications to the seed or the developing plant (Woo et al., 2014), and any positive changes noted by the treatments are observed on the growing plant (biomass) and the developing fruit, which are reflected in the biometric parameters important for evaluating yield quantity. However, only a very few reports have provided information on specific genes or enzymatic activities in the fruits, when the corresponding plants have been inoculated with *T. harzianum* preparations; the only case is for tomato (Chacón et al., 2007; Singh et al., 2018). No dedicated investigations have been performed on fruits collected from plants treated with microbial BCAs in order to evaluate the metabolic pathways and molecular changes that can influence the quality and beneficial health characteristics in the harvested products.

To investigate the outcome of *Trichoderma* on strawberry, in particular on the fruit, this study was undertaken to determine the effects of treatments with different *Trichoderma* strains on the productivity of strawberry, on the plant growth promotion of above/below ground vegetative structures, and the reproductive structures determining fruit yield. Moreover, multiple approaches assayed the physiological characteristics of strawberry after the application of these beneficial fungi to the mother plant, which could have an effect on the corresponding formed fruits, anthocyanin and antioxidant compound content, and the representation of proteins associated with signaling, energetic and metabolic processes, as well as with plant response to biotic/abiotic stresses. This study provides evidences that *Trichoderma* can have positive effects on above-mentioned plant physiological parameters, as well as on beneficial compounds that highly influence food quality and consumer health.

## Materials and Methods

### Fungal Strains

*Trichoderma harzianum* strains T22 and TH1, and *T. virens* strain GV41 were obtained from a microbial collection available at the Department of Agricultural Sciences of the University of Naples Federico II, Portici, Italy, and cultivated bimonthly on Potato Dextrose Agar (HI-MEDIA, Pvt. Ltd., Mumbai, India), at 25°C. *Trichoderma* propagules were produced by solid-state fermentation on sterile rice bran (500 g) inoculated with a spore suspension (1 × 10^6^ spores/mL), and incubated at 25°C. After 7 days, the spores were collected washing the rice bran with sterile water. Spore suspensions were adjusted to the desired concentration by using a haemocytometer. *T. harzianum* strain T-22 and *T. virens* strain G-41 (GV41) are registered mBCA Plant Protection Products, components biofungicides on the agricultural market that meet safety criteria established by EPA and EU governing bodies. *T. harzianum* strain TH1 does not produce noted toxic compounds as determined by metabolomic analysis (Vinale and Woo, data not shown).

### Plant Material, Treatments and Sampling

Experiments were carried out in the greenhouse at the Department of Agricultural Sciences of the University of Naples Federico II, Portici, Italy, under natural, seasonal environmental conditions. Fresh transplants of uniform size of *Fragaria x ananassa* cv. Sabrina were transplanted in October 2016 into 25 cm-diameter pots (one plant per pot) filled with sterile soil. The trial was arranged in a completely randomized block design with 2 biological replicates per treatment and 10 plants in each replicate. Fungal spore suspensions (T22, TH1, and GV41) were tested at 10^7^ spores/mL in water. They were applied once by root dip (15 min) immediately prior to transplanting, and monthly by irrigation (25 mL) until 7 days before the first harvest. Controls consisted in water-treated plants (CTR). Throughout the duration of the field experiment, the disease incidence of the most common strawberry pathogens was monitored on plants and fruits.

Ripe fruits were harvested at the commercial stage from *Trichoderma*-treated and control plants once per week from April to June 2017 and individually counted and weighed. All strawberry plants were harvested at the end of June 2017, and thoroughly washed under running tap water to remove soil particles. Plants were dried in oven at 65°C for about 72 h, until achieving a constant weight. For each treatment, total yield (TY), number of fruits/plant (NF), root length (RL), root fresh weight (RFW), and root dry weight (RDW) were measured.

### Preparation of Fruit Samples for Chemical and Proteomic Analyses

Red ripe fruits from *Trichoderma-*treated and control plants were immediately ground in liquid N_2_ and stored at −80°C until their use. For chemical analyses, individual freeze-dried strawberries were stored in a desiccator, at room temperature, in the dark, and then pulverized by using a knife mill Grindomix GM 200 (Retsch, Haan, Germany). Powdered strawberry samples were pooled according to treatment and used for further chemical analyses.

For proteomic analysis, individual frozen strawberries were pooled according to treatment, grounded in a blender and finally grinded in a mortar, using copious liquid N_2_ to avoid tissue defrosting. Samples were then lyophilized, and immediately processed for further proteomic analysis.

### Determination of Total Antioxidant Capacity and Total Phenolic Content in Fruits

The 2,2-diphenyl-1-picrylhydrazyl (DPPH) free radical assay was used to measure total antioxidant capacity (TAC) of strawberry fruit hydroalcoholic extracts (Sharma and Bhat, 2009). Briefly, 1 mL of a solution 1% v/v formic acid in methanol:water (70:30 v/v) was added to 10 mg of each sample, which was then homogenized; the suspension was vortexed and then centrifuged (14800 rpm, 4°C, for 10 min). DPPH was dissolved in methanol (0.4 mg/mL) and the absorbance at a wavelength of 517 nm was adjusted to 0.9 ± 0.02 by using a T92+ UV double beam spectrophotometer (PG Instruments, Leicester, United Kingdom). Scavenging capacity was evaluated by dissolving 0.2 mL of each hydroalcoholic extract in 0.9 mL of DPPH solution; after incubation at 25°C, for 10 min, the absorbance was measured at 517 nm. Percentage of inhibition was calculated with respect to a solution of 1% v/v formic acid in methanol:water (70:30 v/v). A trolox calibration curve was built in the range 10-120 μM, and TAC was expressed as μmol of trolox equivalent per gram of dry matter. Each extraction was performed in duplicate for a whole of eight observations for each sample.

Total phenolic content (TPC) was measured through the colorimetric Folin-Ciocalteu method, following the procedure of Singleton et al. (1998). Gallic acid was used as a standard, and a series of calibration solutions was prepared in the concentration range 0.020–0.150 mg/mL. Sample hydroalcoholic suspensions were prepared as described above. A 0.1 mL sample aliquot was mixed with 0.5 mL of distilled water and 125 μL of Folin-Ciocalteu solution. The mixture was vortexed (1000 rpm), left for 6 min at room temperature and added with 1.25 mL of 0.70 M sodium carbonate. The mixture was vigorously vortexed and incubated for 90 min, at room temperature. The absorbance of samples was measured at 760 nm.

### Determination of Ascorbic Acid Content in Fruits

Strawberry samples (500 mg) were extracted with 5 mL of an aqueous solution containing 3% v/v metaphosphoric acid and 8% v/v acetic acid. Upon vortexing and centrifugation (4000 rpm, at 4°C, for 10 min), acid extracts were titrated using an indophenol solution (25% w/v 2,6-dichloroindophenol, 21% w/v NaHCO_3_), until a light pink color appeared. Different concentrations of ascorbic acid were titrated with above-mentioned indophenol solution in order to build up a standard calibration curve (Ramirez et al., 1996).

### Analysis of Anthocyanins in Fruits by LC–DAD–ESI-MS/MS

Samples were extracted according to Holzwarth et al. (2012), with minor modifications. Strawberry dried samples (50 mg) were suspended in 3 mL of 5% v/v formic acid in methanol, sonicated for 10 min, at 40°C, and finally placed in a water bath (40°C), under agitation. Samples were centrifuged (4000 rpm, at 4°C, for 10 min), and 1 mL of each supernatant was dried at 40°C in a Savant centrifugal evaporator (Thermo-Fisher, Bremen, Germany). Dried extracts were dissolved in 0.3 mL of 5% v/v formic acid, filtered with modified cellulose filters (0.22 μm, Phenomenex, Torrance, CA, United States), and 10 μL of each solution was injected into the LC system. Quantitative analysis of anthocyanins was performed by using a Shimadzu LC10AD binary system (Shimadzu, Kyoto, Japan) equipped with a SPD-M10A diode array detector (DAD, Shimadzu) and a Series 200 autosampler (Perkin Elmer, Billerica, MA, United States). Chromatographic separation was achieved through a Kinetex XB-C18 column (150 × 4.6 mm, 5 μm, 100 Å, Phenomenex) equipped with a C18 ODS guard column (4.0 × 3.0 mm), at 25°C, with a flow rate of 0.8 mL/min. Mobile phase A was 5% v/v formic acid and mobile phase B was 5% v/v formic acid in methanol. The following binary gradient (min/%B) was used: (0/20), (3/20), (15/55), (18/55), (22/90), (25/90). Typical benzopyrylium and flavylium ions of anthocyanins were monitored at 520 nm.

For peak assignment, samples were injected into an API2000 triple quadrupole tandem mass spectrometer (AB Sciex, Carlsbad, CA, United States) by using the same chromatographic conditions listed above. Positive electrospray ionization was used for the detection; source parameters were as follows: spray voltage 5.5 kV; capillary temperature 300°C, dwell time 100 ms. The chromatographic profile was recorded in multiple reaction monitoring mode (MRM). Tentative identification of individual anthocyanins was achieved according to Määttä-Riihinen et al. (2004) by using mass transitions given in parentheses: cyanidin 3*-O*-glucoside (*m/z* 449 → 287), pelargonidin 3*-O*-glucoside (*m/z* 433 → 271), pelargonidin 3*-O*-rutinoside (*m/z* 579 → 271), pelargonidin 3*-O*-malonyl-glucoside (*m/z* 519 → 271), pelargonidin 3*-O*-acetyl-glucoside (*m/z* 475 → 271) and cyanidin derivative (*m/z* 449 → 287). Individual anthocyanins were quantified using calibration curves of pelargonidin 3*-O*-glucoside, while cyanidin was used for the quantification of anthocyanidins (aglycone form). Three sets of calibration curves were built in the range 0.1–50 μg/mL according to the limit of detection and the limit of quantitation (Armbruster and Pry, 2008). Three replicates of the solutions 50 ng/mL were injected into the LC-DAD system to verify the lowest concentration for which the signal to noise ratio was higher than three. The *r*^2^ value was calculated plotting the area counts against the injected concentrations. Each point of the calibration curves was injected three times in the same day (intraday assay for the repeatability) and three times in three different days (interday assay for the reproducibility); the accuracy was reported as the discrepancies between nine calibration curves performed intraday and interday. Slope among the calibration curves was calculated and compared to each point of each calibration curve. Results were expressed as relative standard deviation (%). Each sample was extracted and injected twice for a total of 4 observations; results were reported as μg/g of fruit sample. In case of lacking standards, the calibration of structurally related compounds was used and corrected by a molecular weight factor (Chandra et al., 2001).

### Fruit Protein Extraction, Digestion and Peptide Fractionation

Lyophilized samples from pooled frozen fruits of *Trichoderma-*treated and control strawberry plants (9 fruits collected from 3 plants for each condition) were quickly extracted in parallel for proteins through a slightly modified phenol-extraction method followed by ammonium acetate–methanol precipitation (Li et al., 2015). Thus, 1 g of lyophilized powder of each sample was mixed in a mortar with 1% w/w polyvinylpyrrolidone, and resuspended in 10 mL of 0.7 M sucrose, 0.1 M KCl, 0.5 M Tris–HCl, 50 mM EDTA, 40 mM DTT, pH 8.5, containing a protease inhibitors cocktail for plant tissues (Sigma-Aldrich, United States). Homogenization was performed in an Ultra-Turrax tissue processor (IKA, Werke GmbH, Germany) for 1 min, at 6000 rpm. Tris-buffered phenol, pH 8.0 (Sigma-Aldrich) was added to the suspension (1:1, v/v) and the sample was mixed thoroughly and centrifuged at 10,000 × *g*, for 15 min, at 4°C. The extraction was repeated twice and phenol phases were collected and precipitated with 5 vol of cold 0.1 M ammonium acetate in methanol, at −20°C, overnight. Protein pellets were washed twice with ice-cold methanol and finally with cold acetone containing 20 mM DTT, and then air-dried. Protein pellets (5 mg) were solubilized in 250 μL of 7 M urea, 2 M thiourea, 50 mM triethylammonium bicarbonate (TEAB), 2% SDS, 10 mM DTT, pH 8.5, and added with plant specific protease inhibitors (Sigma-Aldrich). Samples were vortexed and incubated for 1 h, at 30°C, under shaking. Samples were centrifuged at 12,000 × *g* for 5 min, at 5°C, and the taken of the supernatant containing the corresponding protein extract. Protein concentration was determined using the Bio-Rad Protein Assay (Bio-Rad, Hercules, CA, United States), according to manufacturer’s instructions. Relative quantification of individual proteins was obtained by a tandem mass tagging (TMT)-labeling experiment using a TMT10plex Isobaric Label Reagent Kit (Thermo-Fisher Scientific, United States); protein samples were prepared according to manufacturer’s instructions. Thus, an aliquot of each protein sample (100 μg) was adjusted to a 100 μL final volume with 100 mM TEAB, and then treated as reported in the manufacturer’s instructions. Each sample was digested with freshly prepared trypsin (enzyme to protein ratio 1:50 w/w) in 100 mM TEAB, at 37°C, overnight. Resulting peptides from each protein sample were labeled with the TMT10plex Label Reagent Set (Thermo-Fisher Scientific), at 25°C, following manufacturer’s instructions, according to the labeling scheme: Ctrl-TMT10-126, T22-2-TMT10-127N, TH1-TMT10-127C, and GV41-TMT10-128N. After 1 h of reaction, 8 μL of 5% w/v hydroxylamine was added in each tube and mixed for 15 min, in order to quench the derivatization reaction. For a set of comparative experiments, tagged peptide mixtures were mixed in equal-molar ratios (1:1:1:1) and vacuum-dried under rotation. Then, pooled TMT-labeled peptide mixtures were suspended in 0.1% v/v trifluoroacetic acid, and fractionated by using the Pierce High pH Reversed-Phase Peptide fractionation kit (Thermo-Fisher Scientific) according to manufacturer’s instructions. After fractionation, eight fractions of TMT-labeled peptides were collected, vacuum-dried and finally reconstituted in 0.1% v/v formic acid for subsequent mass spectrometric analysis.

### NanoLC-ESI-Q-Orbitrap MS/MS Analysis of Fruit Protein Digests

Tandem mass tagging-labeled peptide fractions were analyzed with a nanoLC-ESI-Q-Orbitrap-MS/MS platform consisting of an UltiMate 3000 HPLC RSLC nano system (Dionex, United States) coupled to a Q Exactive Plus mass spectrometer through a Nanoflex ion source (Thermo-Fisher Scientific). Peptides were loaded on an Acclaim PepMap RSLC C18 column (150 mm × 75 μm ID, 2 μm particles, 100 Å pore size, Thermo-Fisher Scientific), and eluted with a gradient of solvent B (19.92/80/0.08 v/v/v water/acetonitrile/formic acid) in solvent A (99.9/0.1 v/v water/formic acid), at a flow rate of 300 nL/min. The gradient of solvent B started at 5%, increased to 60% over 125 min, raised to 95% over 1 min, remained at 95% for 8 min. The mass spectrometer operated in data-dependent mode, using a full scan (*m/z* range 375–1500, nominal resolution of 70,000), followed by MS/MS scans of the 10 most abundant ions. MS/MS spectra were acquired in a scan *m/z* range 110–2000, using a normalized collision energy of 32%, an automatic gain control target of 100,000, a maximum ion target of 120 ms, and a resolution of 17,500. A dynamic exclusion value of 30 s was also used.

### Bioinformatics for Protein Identification and Quantitation

Raw data files were analyzed for protein identification and relative protein quantification with Proteome Discoverer v. 2.1 software (Thermo Scientific), enabling the database search by Mascot algorithm v. 2.6 (Matrix Science, United Kingdom) using the following criteria: NCBI protein database (Viridiplantae, 6216064 protein sequences, 12/2018) including the most common protein contaminants; carbamidomethylation of Cys and TMT modification of lysine and peptide N-terminal as fixed modifications; oxidation of Met, deamidation of Asn and Gln, pyroglutamate formation of Gln as variable modifications. Peptide mass tolerance was set to ±10 ppm and fragment mass tolerance to ±0.02 Da. Proteolytic enzyme and maximum number of missed cleavages were set to trypsin and 2, respectively. Protein candidates assigned on the basis of at least 2 sequenced peptides and an individual Mascot Score ≥ 30 were considered as confidently identified. For quantification, ratios of TMT reporter ion intensities in the MS/MS spectra from raw datasets were used to calculate fold changes between samples. Results were filtered to 1% false discovery rate. Proteomic data have been deposited to the ProteomeXchange consortium (Vizcaíno et al., 2016) with the PRIDE partner repository with the dataset identifier PXD016951.

### Bioinformatics for Protein Functional Analysis

Identified proteins were subjected to BLAST sequence homology search using command line NCBI applications against the *Arabidopsis thaliana* protein sequence database TAIR 10 from The Arabidopsis Information Resource repository^2^. Functional categorization of differentially represented proteins (DRPs) was obtained using Mercator pipeline^3^ for automated sequence annotation. Final outputs were integrated with data from available literature. Hierarchical clustering analysis of log_2_ transformed abundance ratios of DRPs from *Trichoderma-*treated strawberry plants was performed using Genesis 1.8.1 platform (Sturn et al., 2002). Person’s correlation as distance and average linkage clustering were chosen as parameters. Protein interaction networks were obtained with STRING v. 11^4^ using the *A. thaliana* database. Venn diagrams were depicted using a web tool at http://bioinformatics.psb.ugent.be/webtools/Venn.

### Statistical Analysis

Biometric data (TY, NF, RL, RFW, and RDW), and total antioxidant capacity, ascorbic acid, total phenolic compounds, total anthocyanins and single anthocyanin content of samples were examined by one-way ANOVA using SPSS software (v.15.0 IBM, Armonk, NY, United States). Significant differences among treatments were separated using SNK (Student–Newman–Keuls) and Fisher’s Least Significant Difference (LSD) *post hoc* tests, at the 0.05 level of significance.

## Results

### Strawberry Growth and Yield of Strawberry Plants

Treatments with *Trichoderma harzianum* strains T22 and TH1, and *T. virens* strain GV41 significantly enhanced total yield (TY) (*P* < 0.05) of strawberry plants, as compared to control (CTR) (Table 1). In particular, the strains T22, TH1, and GV41 increased TY by 35, 38, and 29%, respectively. Applications of *T. harzianum* strains T22 and TH1 were also found to significantly increase (*P* < 0.05) the number of fruits per plant (NF) by 17 and 39%, respectively, while a lower effect (6%) was observed in the case of *T. virens* strain GV41.

**TABLE 1:** Effects of different *Trichoderma* strains (T22, TH1, and GV41) on the growth and productivity of strawberry plants under greenhouse conditions.

**Treatment**	**Total Yield (TY) (g/plant)**	**Number of fruits/plant (NF)**	**Root length (RL) (cm/plant)**	**Root fresh weight (RFW) (g/plant)**	**Root dry weight (RDW) (g/plant)**
	
	**Mean ± SD (%)**	**Mean ± SD (%)**	**Mean ± SD (%)**	**Mean ± SD (%)**	**Mean ± SD (%)**
CTR	125.4 ± 21.8 a	6.4 ± 1.2 ab	22.0 ± 1.9 a	62.9 ± 5.4 ab	13.5 ± 1.1 bc
T22	168.9 ± 25.2 b (35)	7.5 ± 1.2 c (17)	24.5 ± 2.2 b (11)	69.3 ± 7.8 bcd (10)	15.1 ± 1.3 cd (12)
TH1	173.1 ± 24.2 b (38)	8.9 ± 1.2 d (39)	24.0 ± 3.2 ab (9)	64.8 ± 7.7 abc (3)	15.1 ± 1.8 cd (12)
GV41	161.6 ± 24.1 b (29)	6.8 ± 1.2 bc (6)	24.5 ± 2.7 b (11)	73.7 ± 5.4 d (17)	16.4 ± 1.5 a (21)

*Treatments were applied at the time of transplant (by root dip) and monthly by irrigation. Data represent the mean value of 10 biological replicates ± standard deviation (SD). Different letters in a single column indicate statistically significant differences for *P* < 0.05. Increments or decrements compared to control (CTR) are shown in percent (%) and are reported in parenthesis.*

All *Trichoderma* treatments slightly enhanced root length (RL), root fresh weight (RFW) and root dry weight (RDW), and GV41 showed the highest increase of RL (11%), RFW (17%) and RDW (21%) compared to CTR, as outlined in Table 1.

In addition, no incidence of disease was observed on the plants or fruits subjected to the *Trichoderma* biological treatments monitored for the duration of the experiment (data not shown).

### Antioxidant Properties of Strawberry Fruits

Treatments with *Trichoderma* strains variably affected the antioxidant capacity of fruits, as well as the corresponding total polyphenol, ascorbic acid and total anthocyanin content. In particular, application of the strain GV41 exhibited a slight increase (8%) of antioxidant activity in strawberry samples respect to CTR (*P* < 0.05), together with a significant accumulation of ascorbic acid (23%) and total anthocyanins (31%) (*P* < 0.05) (Table 2). Conversely, strain TH1 only promoted a significant increase (66%) of total anthocyanin levels (*P* < 0.05).

**TABLE 2:** Effects of the application of different *Trichoderma* strains (T22, TH1, and GV41) on the antioxidant properties of strawberry fruits.

**Treatment**	**Antioxidant capacity [μmol eq Trolox/g]**	**Total polyphenols [mg/g]**	**Ascorbic acid [mg/100g]**	**Total anthocyanins [μg/g]**
	
	**Mean ± SD (%)**	**Mean ± SD (%)**	**Mean ± SD (%)**	**Mean ± SD (%)**
CTR	54.0 ± 10.7 bc	10.9 ± 0.1 a	116.8 ± 12.6 bc	809.0 ± 13.0 bc
T22	47.9 ± 0.9 abc (−11)	8.6 ± 2.9 a (−21)	107.0 ± 13.1 b (−8)	818.1 ± 23.5 bc (1)
TH1	42.8 ± 16.7 a (−21)	9.5 ± 8.4 a (−13)	102.8 ± 30.8 ab (−12)	1340.7 ± 15.6 a (66)
GV41	58.3 ± 0.3 c (8)	10.4 ± 2.5 a (−5)	144.2 ± 2.5 d (23)	1056.6 ± 1.9 b (31)

*Treatments were applied at the transplant (root dip) and monthly by irrigation. Data represent the mean value of eight biological replicates ± standard deviation (SD). Different letters in a single column indicate statistically significant differences for *P* < 0.05. Increments or decrements compared to control (CTR) are reported as % values in parenthesis.*

### Quali-Quantitative Characterization of Individual Anthocyanins in Strawberry Fruits

To better evaluate anthocyanin content in strawberry fruits from *Trichoderma*-treated plants, a dedicated quali-quantitative characterization of individual compounds was undertaken. To this purpose, mass spectrometry (MS) transitions were tentatively identified and assigned according to previous studies (Chandra et al., 2001; Määttä-Riihinen et al., 2004; Holzwarth et al., 2012); the results are reported in Supplementary Table S1, while Supplementary Figure S1 depicts a representative anthocyanin profile of a fruit sample recorded at 520 nm. Peaks 1 and 6 were assigned to cyanidin 3*-O*-glucoside (cya 3*-O*-glc) and cyanidin derivative (cya der), respectively; both compounds exhibited an [M^+^] signal at *m/z* 449, releasing cyanidin as fragment at *m/z* 287 in MS/MS experiments. As expected based on chromatographic gradient and cationic selectivity of the column, cya 3*-O*-glc eluted earlier than cya der. Peaks 2, 3, 4, and 5 were tentatively assigned to pelargonidin glycosides since each component showed the flavylium ion signal at *m/z* 271 (pelargonidin flavylium ion) in MS/MS experiments. In agreement with previous investigations (Holzwarth et al., 2012), the predominant anthocyanin (peak 2) was pelargonidin 3*-O*-glucoside (pel 3*-O*-glc) with a [M^+^] signal at *m/z* 433. On the other hand, peak 3, 4 and 5 were assigned to pelargonidin 3*-O*-rutinoside (pel 3*-O*-rut), pelargonidin 3*-O*-malonyl-glucoside (pel 3*-O*-mal-glc) and pelargonidin 3*-O*-acetyl-glucoside (pel 3*-O*-ac-glc) based on corresponding [M^+^] signals at *m/z* 579, 519 and 475, respectively.

**FIGURE 1:**
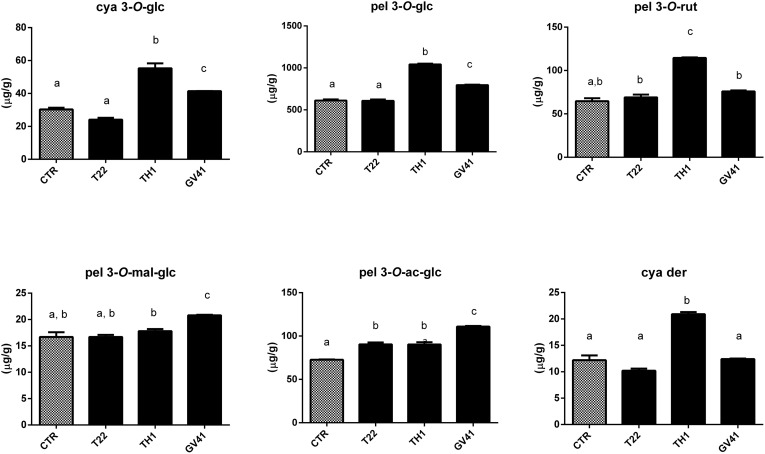
Concentration of individual anthocyanins in strawberry fruits produced by plants subjected to the treatment with *Trichoderma* strains (T22, TH1, and GV41), as compared to control (CTR). Results on cyanidin 3*-O*-glucoside (cya 3*-O*-glc), pelargonidin 3*-O*-glucoside (pel 3*-O*-glc), pelargonidin 3*-O*-rutinoside (pel 3*-O*-rut), pelargonidin 3*-O*-malonyl-glucoside (pel 3*-O*-mal-glc), pelargonidin 3*-O*-acetyl-glucoside (pel 3*-O*-ac-glc) and cyanidin derivative (cya der) are shown. Data were reported as μg/g sample, and represent the mean value of 8 biological replicates ± standard deviation (SD). Different letters on the bars indicate statistically significant differences (*P* < 0.05).

Based on above-mentioned results, LC–DAD-assisted quantitative measurements of individual anthocyanins in fruits from plants exposed to the *Trichoderma* spore suspensions (Figure 1) confirmed the increment of total compounds already detected by colorimetric test (Table 2). The highest increases were observed with the application of strain TH1, which promoted the accumulation of cya 3*-O*-glc (82%), pel 3*-O*-glc (70%), pel 3*-O*-rut (77%), pel 3*-O*-ac-glc (24%) and cya der (72%) (*P* < 0.05), compared to CTR; no significant differences were envisaged for pel 3*-O*-mal-glc. Similarly, the strain GV41 increased the accumulation of all the individual anthocyanins (*P* < 0.05), with the exception of cya der. Finally, the application of strain T22 only determined a 24% increase in pel 3*-O*-ac-glc content (*P* < 0.05).

### Proteomic Analysis of Strawberry Fruits

**FIGURE 2:**
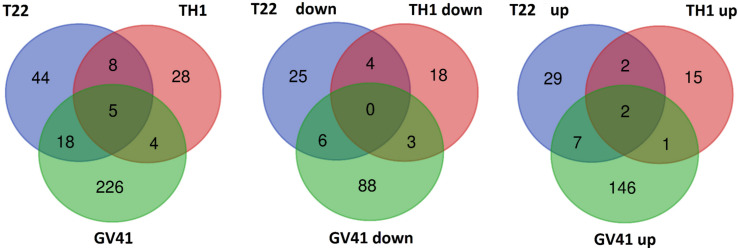
Venn diagram showing differentially represented proteins present in strawberry fruits produced by plants subjected to the treatment with *Trichoderma* strains (T22, TH1, and GV41), as compared to control. Diagrams refer to all differentially represented proteins **(left)**, those down-represented **(middle)** and over-represented **(right)**, respectively.

**FIGURE 3:**
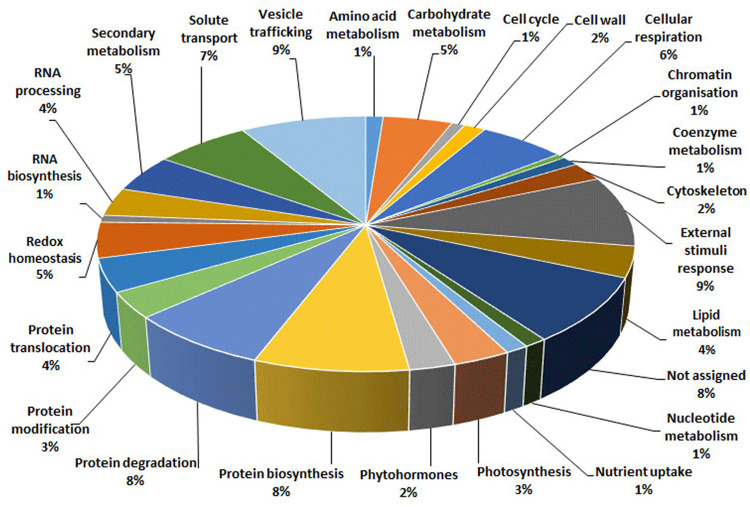
Functional distribution of differentially represented proteins present in strawberry fruits produced by plants subjected to the treatment with *Trichoderma* strains (T22, TH1, and GV41), as compared to control (CTR). Identified protein species were initially assigned with Mercator software (Supplementary Table S3), followed by a functional group cataloguing including information from the Bevan classification (Bevan et al., 1998) and recent literature data.

**FIGURE 4:**
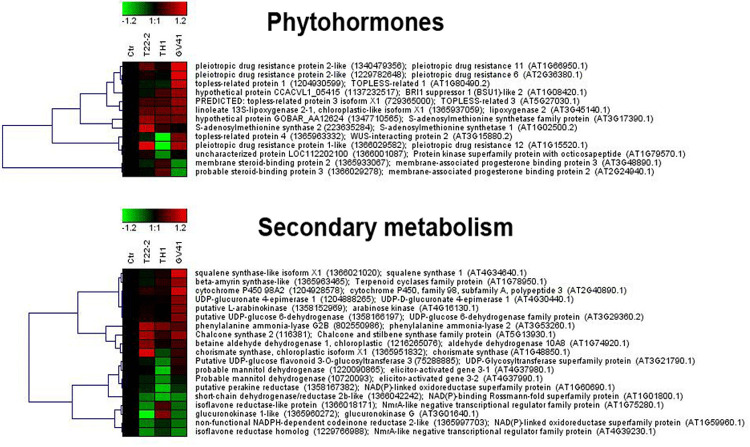
Heat-map representation and hierarchical clustering analysis of proteins related to phytohormone metabolism **(upper panel)** and secondary metabolism **(lower panel)**, which were differentially represented in strawberry fruits produced by plants subjected to the treatments with *Trichoderma* strains (T22, TH1, and GV41), as compared to control (CTR). Proteins shown are those with abundance fold changes ≥1.50 or ≤0.66 compared to control (*P* ≤ 0.05) (Supplementary Table S3). Data are reported as log_2_ transformed abundance ratio values. Hierarchical clustering analysis of DRPs was performed using Genesis 1.8.1 platform (Institute for Genomics and Bioinformatics, Graz University of Technology).

With the aim of evaluating molecular effectors/metabolic pathways that underlie the above-mentioned effects of *Trichoderma* spp. on strawberry fruits, and obtaining original information on fruits from a plant treated with BCAs, protein extracts from berry samples of plants inoculated with *Trichoderma* strains T22, TH1, and GV41 were comparatively evaluated to the control by a TMT-based proteomic approach. This quantitative analysis allowed the identification of 3294 proteins and measuring the relative quantitative levels of 3014 plant proteins (data available in PRIDE repository with dataset identifier PXD016951) that found a counterpart in 3262 and 2982 redundant *Arabidopsis thaliana* entries in TAIR 10 database, respectively, plus additional 32 plant sequence entries not having a homolog in the same sequence data inventory. This proteomic analysis of the strawberry did not note the presence of *Trichoderma* proteins in the fruit extracts. Above-mentioned proteins were further filtered for abundance fold changes ≥1.50 or ≤0.66 (T22, TH1, and GV41 *vs*. CTR) (*P* ≤ 0.05) and accession redundancy, thus ascertaining 333 differentially represented proteins (DRPs) associated with various *Trichoderma* treatments (Supplementary Table S2). The latter corresponded to 323 non-redundant *A. thaliana* sequence entries in TAIR 10 database plus additional 10 plant sequence entries not having an *A. thaliana* counterpart. In particular, 75, 45, and 253 DRPs were observed for *Trichoderma* strains T22, TH1, and GV41, respectively. A Venn diagram representation of these DRPs showed a number of unique and shared components between different *Trichoderma* treatments (Figure 2). Hierarchical clustering of abundance ratios and distribution of DRPs between different *Trichoderma* strains highlighted that most significant quantitative changes occurred after treatment with *T. virens* GV41, followed by that with *T. harzianum* T22 and TH1 (data not shown and Figure 2). Resultant DRPs were functionally indexed through an initial assignment obtained with Mercator software, followed by a functional group cataloguing including information from the Bevan classification (Bevan et al., 1998) and recent literature data (Figure 3 and Supplementary Table S3). This analysis attributed a function to all proteins, except 23 molecular species that were not assigned to any known ontology or functional group. Thus, DRPs were mostly related to the functional category of protein metabolism (including components involved in protein biosynthesis, protein degradation and protein translocation) (20%), stress response (including components associated with redox homeostasis, external stimuli response and protein modification) (17%), carbon and energy metabolism (including enzymes related to carbohydrate metabolism, energy and photosynthesis) (14%), vesicle trafficking (9%), and secondary metabolism (including enzymes catalyzing biosynthesis/degradation of secondary metabolites and phytohormones) (7%), thus highlighting prominent molecular mechanisms and metabolic pathways modified following *Trichoderma* treatments. No major differences in the functional distribution were observed when DPRs from plants treated with strains T22, TH1, and GV41 were considered singularly (data not shown). Functional enrichments of above-mentioned proteins for biological processes, and molecular functions and KEGG pathways confirmed the involvement of most DRPs in the response to different chemical stimuli, in binding to ions/small molecules and catalytic activity, or in the biosynthesis of secondary metabolites, oxidative phosphorylation, and carbon and protein metabolism, respectively (Supplementary Table S4). Heat-map pictures originated from hierarchical clustering of quantity ratios of DRPs for each functional group were reported in Figure 4 and Supplementary Figures S2–S9. These figures describe the relative quantitative representation profile of the different strawberry proteins as result of the different *Trichoderma* treatments, as related to CTR. A general coherent qualitative trend of DRPs between the various treatments with *Trichoderma* strains was observed, with a very limited number of exceptions. In the subsequent sections focused on the most represented protein functional groups, these heat-map pictures are discoursed together with corresponding DRPs and metabolic pathways/molecular processes.

**FIGURE 5:**
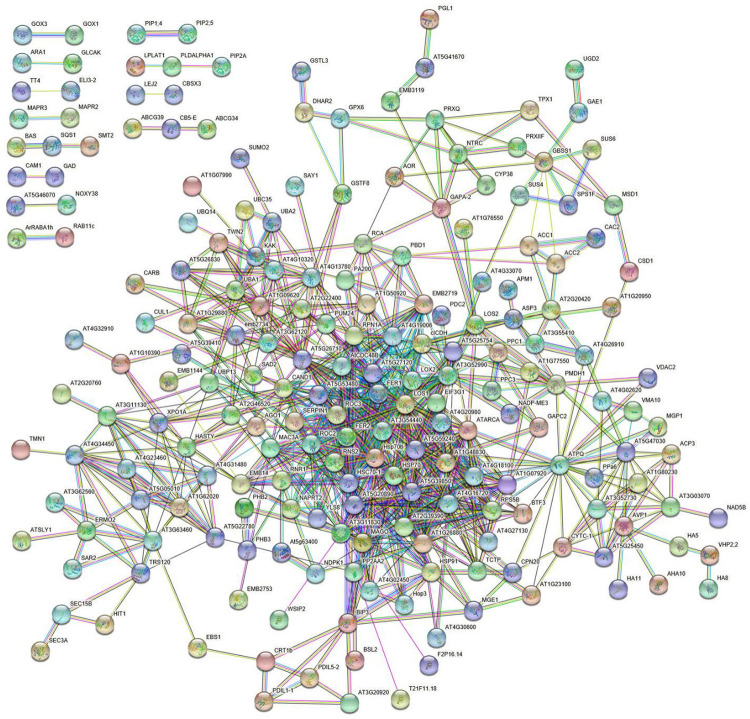
STRING analysis of differentially represented proteins present in strawberry fruits produced by plants subjected to the treatments with *Trichoderma* strains (T22, TH1, and GV41), as compared to control (CTR). Functional protein associations were based on data recorded for *A. thaliana* protein homologs. Only high-confidence interactions (0.7) are shown. Protein codes are reported in Supplementary Table S5.

Bioinformatic analysis of DRPs with STRING allowed predicting a strawberry functional protein association map based on *A. thaliana* homolog counterparts, which at high confidence (0.7) revealed a predominant highly ramified network linking together 182 components, plus twelve binary/ternary molecular complexes (Figure 5 and Supplementary Table S5). The involvement of most DRPs (54.6% of total number) in this major network emphasized the occurrence of a functional assembly bridging different deregulated metabolic pathways and molecular processes, which underlies the physiological adaptation of strawberry fruits to the occurrence of *Trichoderma* strains in plant roots. As mentioned above, most of the knots present in this network were associated with DRPs from treatment with *T. virens* GV41. When a medium confidence (0.4) was used, a unique highly ramified network linking together 287 DRPs (86.2% of their total number) was observed (data not shown). Overall, above-mentioned proteomic results suggested that various metabolic, energetic and signaling processes, together with a number of plant external stimuli-/stress-responsive mechanisms, are simultaneously regulated in strawberry fruits as result of the plant treatment with different *Trichoderma* strains.

## Discussion

Fungi belonging to the genus *Trichoderma* are used as successful plant growth enhancers, biostimulants, biofertilizers, and as effective biocontrol agents against various pathogens (Woo et al., 2014; Lorito and Woo, 2015). Some of these positive effects have been related to the microbial release of bioactive metabolites and elicitor proteins in the plant rhizosphere (Harman et al., 2004a, b; Vinale et al., 2014). Proteomic and transcriptomic studies on plant root, leaf tissues, and seedlings suggested that this growth-promoting activity of *Trichoderma* spp. is associated with different molecular events in the host, such as: (i) an increased nutrient uptake (Altomare et al., 1999); (ii) variations in phytohormone levels and corresponding induced metabolic processes (Harman et al., 2004a; Caporale et al., 2014); (iii) a general augmented carbon and energy metabolism (Shoresh et al., 2010); (iv) a higher photosynthetic efficiency (Shoresh et al., 2010). Molecular data on fruit tissues are scant in the literature and mainly limited to specific genes/proteins/metabolites (Singh et al., 2018).

### Growth Promotion and Related Molecular Mechanisms

This study on strawberry plants and fruits confirmed the ability of the selected *T. harzianum* (T22 and TH1) and *T. virens* (GV41) strains to act as growth-promoting agents. In fact, these treatments increased significantly strawberry plant total yield (from a value of 29% for GV41 to 38% for TH1), augmented the number of corresponding fruits (from a value of 6% for GV41 to 39% for TH1) and promoted corresponding root growth (from a value of 12% for TH1 and T22 to 38% for GV41), when compared to control.

It has already been reported that the yield in strawberry plants is strongly dependent on micronutrient availability in the soil (Valentinuzzi et al., 2015). Our proteomic results on strawberry fruits confirmed the positive effect of *Trichoderma* strains in facilitating plant nutrient uptake, as demonstrated by the observed over-representation of membrane proteins devoted to (neutral and ionic) solute transport (Supplementary Table S4) and, in general, by the functional enrichment of biological processes related to the response to various chemicals (Supplementary Table S4). This phenomenon was more evident for GV41, but also detectable in T22 and TH1. Among the above-mentioned augmented proteins, worth mentioning are plasma membrane intrinsic proteins 1;4, 2, 2A and 2;5 allowing the cellular import of water and small neutral molecules (Wang et al., 2016), as well as membrane transporters for ATP/ADP, malate, nucleotide derivatives, and xanthine/uracil/ascorbate (Supplementary Figure S2). The selective over-representation of the latter protein in *Trichoderma* GV41-treated plants may be related to the augmented levels of ascorbic acid measured in the corresponding fruit (Table 2). In the above-mentioned context, it could also be considered the observed over-representation of five H^+^-translocating and two Ca^2+^-translocating ATPases, which couple the well-known ion fluxes associated with plant response to BCAs (Shoresh et al., 2010) to energy production (Supplementary Figure S2). Additional over-represented membrane components devoted to phytohormone, oxidant and proton translocation were ABCG transporters (3 pleiotropic drug resistance proteins) (see below) and two H^+^-exporting pyrophosphatases. The latter proteins have been widely recognized to play an essential role in auxin-mediated leaf/fruit development, biomass accumulation and crop yield increase (Li et al., 2005; Schilling et al., 2014; Yang et al., 2014). On the other hand, the observed down-representation of the nutrient-related chloroplast nitrogen regulatory protein PII (GlnB) in fruits from treated plants was in line with previous observations on the reduced transcription of the corresponding gene in the presence of elevated levels of N-containing metabolites (Hsieh et al., 1998; Supplementary Figure S2). GlnB also played an essential role in regulating target proteins in response to cellular ADP/ATP levels and 2-oxoglutarate status, thereby coordinating the plant carbon/nitrogen balance and regulating specific metabolic pathways (Uhrig et al., 2009). In the whole, our gel-free proteomic approach revealed abundance changes of membrane nutrient transporter proteins already ascertained by transcriptomic studies (De Palma et al., 2016, 2019), but whose solubility hampered corresponding detection in previous gel-based methods.

In addition to above-mentioned H^+^- and Ca^2+^-translocating ATPase machineries, proteomic analysis of strawberry fruits also provided information on additional mechanisms ensuring the greater energy supply requested in *Trichoderma*-treated plants to sustain the observed plant growth and development. Thus, different proteins involved in carbohydrate (starch, sucrose and nucleotide-sugar) metabolism (β-galactosidase, β-fructofuranosidase, granule-bound starch synthase 1, sucrose synthase 1F, 4 and 6, xyloglucan 6-xylosyltransferase, UDP-glucuronate 4-epimerase 1, glucuronokinase 1, UDP-glucose 6-dehydrogenase, sorbitol dehydrogenase and arabinose kinase), glycolysis and tricarboxylic acid cycle (two phosphofructokinase isoforms, pyruvate kinase, isocitrate dehydrogenase, ATP lyase and 2-oxoglutarate dehydrogenase), and alcoholic fermentation (two pyruvate decarboxylase isoforms) showed augmented levels in fruit after *Trichoderma* treatments (Supplementary Figure S3). This effect was again more evident in *Trichoderma* GV41-treated plants, confirming the already-mentioned strain-dependent potential of BCAs (Tucci et al., 2011; Fiorentino et al., 2018; Marra et al., 2019). These results were in good agreement with previous proteomic observations on root and leaf tissues from maize, tomato, cucumber and grapevine plants (Segarra et al., 2007; Shoresh and Harman, 2008; Shoresh et al., 2010; Palmieri et al., 2012; Manganiello et al., 2018). Conversely, all mitochondrial components of the mitochondrial ATP synthase and cytochrome C reductase complexes showed reduced levels after microbial treatments (Supplementary Figure S3), while a mixed behavior was observed for proteins involved in photosynthesis. The first phenomenon may be related to a cell attempt to limit generation of reactive oxygen species (ROS) side products during oxidative phosphorylation, in a physiological plant condition where plant cells are already exposed to significant oxidant fluxes (see below) (Shoresh et al., 2010). On the other hand, components of the NADH dehydrogenase complex (complex I), which is believed to belong to the minimal assembly required for the transfer of electrons from NADH to the respiratory chain, showed augmented levels after *Trichoderma* treatments.

Observed plant growth and development in *Trichoderma*-treated plants also corresponded to increased fruit representation levels of a number of components present in protein biosynthetic machineries involved in: (i) RNA biosynthesis and processing (2 and 8 in number, respectively); (ii) production of amino acid-tRNAs (8 in number); (iii) assembling of large/small ribosomal subunits (8 in number); (iv) polypeptide chain translation initiation/elongation activities (translation initiation factor 3 subunits G and L, and elongation factor G); (v) protein translocation from cytoplasm to cell nucleus and endoplasmic reticulum (3 and 7 in number, respectively) (Supplementary Figures S6, S7). Sporadic proteins showing an opposite quantitative trend were also observed. Above-mentioned components seemed essential to fuel novel enzymes and structural proteins to developing fruit cells and growing tissues (Shoresh and Harman, 2008), as revealed by the number of over-represented species mentioned in the previous paragraphs and the augmented levels of constitutive elements present in evolving cell wall (5 in number) and cytoskeleton (5 in number) compartments (Supplementary Figure S8). In the latter group can also be included the cell division protein FtsZ that, together with all other strawberry deregulated components present in the cell cycle catalog, also showed augmented levels after *Trichoderma* treatment. This protein is a structural homolog of tubulin and mediates ring formation at the chloroplast division site, and thus is essential for plant growth (Vitha et al., 2001). A request for augmented protein levels in treated plants has also to be considered in light of the need of additional defensive components (see below).

### Secondary Metabolites and Related Biosynthetic Pathways

This study also showed that *Trichoderma* spp. treatment of plants favored accumulation of anthocyanins and other antioxidants in strawberry fruits, with a pattern that was strain-dependent. In particular, application of two microorganisms increased significantly total content of anthocyanins (31 and 66% for GV41 and TH1, respectively), ascorbic acid (23% for GV41) and corresponding antioxidant capacity (8% for GV41). Chromatographic measurements of individual anthocyanins confirmed the above-mentioned total increment, highlighting the accumulation of all measured compounds except pel 3*-O*-mal-glc and pel 3*-O*-ac-glc in TH1 and GV41, respectively. Induction of antioxidant metabolites in plant tissues of BCA-treated plants has already been reported; for example, *T. harzianum* inoculation was described to increase polyphenolic content and antioxidant activity in grape (Pascale et al., 2017), as well as GSH/GSSG and ASA/DHA ratios in tomato seedlings (Mastouri et al., 2012). Similarly, *T. asperellum* root colonization of cucumber plants augmented total antimicrobial polyphenols (Yedidia et al., 2003). Our proteomic measurement of enzymes involved in biosynthesis of secondary metabolites provided a rationale to the above-mentioned increase of anthocyanins in strawberry fruits. Indeed, it demonstrated augmented levels of phenylalanine-ammonia lyase (PAL) and chorismate synthase, which control the first reaction steps of the phenylpropanoid pathway yielding phenolic compounds (including anthocyanins), and other enzymes (chalcone and stilbene synthase, UDP-glucuronate 4-epimerase, glucokinase, UDP-glucose 6-dehydrogenase, arabinose kinase) assisting the conversion of corresponding intermediates into final compounds, or forming UDP-sugar moieties to be included into corresponding structures (Figure 4). To increase anabolic efficiency toward anthocyanin production, proteins (elicitor-activated gene 3-1 and 3-2, and two isoflavone reductases) catalyzing transformation of above-mentioned secondary metabolites into lignin derivatives were also down-represented. These results well resembled the augmented PAL levels already reported in roots and leaves of other *Trichoderma*-treated plants (Shoresh and Harman, 2008; Shoresh et al., 2010; De Palma et al., 2016).

Additional deregulated secondary metabolism enzymes showing augmented levels after *Trichoderma* application were squalene synthase and β-amyrin synthase, which are involved in the biosynthesis of sesquiterpenoids/triterpenoids (Figure 4). Terpenoids represent important constituents of herbivore-induced plant volatiles that deter herbivores and/or attract their predators (Sharma et al., 2017). They serve as airborne signals that can induce defense responses in systemic undamaged parts of the plant, and prime defense responses in neighboring plants. Our determinations were in good agreement with those on corresponding metabolic pathways and metabolites in tomato plants challenged with *T. harzianum* (Manganiello et al., 2018), confirming that fungal treatment can influence the plant defensive volatilome.

### Antioxidant Enzymes and Related Molecular Processes

Augmented production of antioxidant metabolites in strawberry fruits may occur as consequence of the induction of different defense mechanisms (Contreras-Cornejo et al., 2011), which follow plant interactions with specific BCAs or pathogens. For example, expression of *Pal* genes is induced by jasmonic acid (JA)/ethylene (ET) signaling during plant defense response (Shoresh et al., 2010), and augmented levels of anthocyanins have already been reported to attenuate the effects of ROS produced to generate an hostile environment in plant tissues after microorganism challenge (Figueroa-Balderas et al., 2006; Senthil-Kumar and Mysore, 2010; Mathys et al., 2012). In particular, it has already been demonstrated that plant co-cultivation with *T. asperellum* increased anthocyanin production in *A. thaliana* leaves (Contreras-Cornejo et al., 2011). Our proteomic experiments showed that this augmented production of antioxidants in fruits from treated plants corresponded to a widespread down-representation of enzymes limiting the detrimental effects of ROS (15 in number and corresponding to different redox protective machineries) (Supplementary Figure S4). These findings apparently contrast with previous observations on root and leaf tissues of *Trichoderma*-challenged plants, which report an over-representation of antioxidant and toxicant-scavenging enzymes therein [Segarra et al., 2007; Shoresh and Harman, 2008; Shoresh et al., 2010; Mastouri et al., 2012; De Palma et al., 2016, 2019; Perazzolli et al., 2016; Zhang et al., 2017; Manganiello et al., 2018), although in some cases a coherent quantitative trend was also described (Vitti et al., 2015; Rubio et al., 2017). Thus, our results on this antioxidant metabolite-rich fruit suggested that either the increased concentration of these small protective compounds after fungal challenge was enough to remodulate ROS levels, without the need of augmented antioxidant protein machineries that are repressed to save plant energy, or the biosynthesis of the latter components was selectively down-regulated to allow high basal levels of detrimental oxidants to contrast bacterial challenge. Future investigations are required in this context.

### Defense Processes and Related Molecular Mechanisms

Proteomic analysis of *Trichoderma*-treated plants also highlighted defensive signaling pathways and corresponding down-stream mechanisms modulated following fungus-plant interaction; a number of them have already been reported to prevent pathogen colonization (Shoresh et al., 2010; Pérez and Goossens, 2013; Guerreiro et al., 2016; Yuan et al., 2017; Aldon et al., 2018; Yu et al., 2018). In particular, signaling pathways involving Ca^2+^, JA, ET and brassinosteroid (BR) effectors appear to be modulated in strawberry fruits following the interaction with *Trichoderma*. This was suggested from the observed modified levels of: (i) a number of Ca^2+^-binding sensors and a MAP3K (10 in number, all showing down-representation except coherent Ca^2+^-translocating ATPase concentrations ensuring ion expulsion from the cell); (ii) lipoxygenase 2 involved in JA biosynthesis (showing over-representation); (iii) S-adenosylmethionine synthase 1 and 2 involved in ET biosynthesis (showing over-representation); (iv) BR-related membrane-associated progesterone-binding proteins 2 and 3, and BRI suppressor 1 and 2 (showing a mixed but coherent quantitative trend); and (v) topless-related protein 1, 2 and 3 (Figure 4 and Supplementary Figures S2, S7). Some proteins mentioned above (especially those sensitive to Ca^2+^) are involved in signal decoding and transduction mechanisms leading to a rapid down-stream activation of cellular ROS burst, protein phosphorylation and transcriptional reprogramming events, as well as to the biosynthesis of defense-related phytohormones JA, ET, and salicylic acid (SA). A number of these quantitative protein changes were in agreement with those already determined in other proteomic/transcriptomic studies on root/leaf tissues from *Trichoderma*-treated maize, tomato and cucumber plants (Segarra et al., 2007; Shoresh et al., 2010; Manganiello et al., 2018; Nogueira-Lopez et al., 2018; De Palma et al., 2019). Notwithstanding this investigation strongly suggested the activation of JA- and ET-mediated defense processes also in fruits from *Trichoderma*-treated strawberry plants, a real comprehension of those involving Ca^2+^ and BR action will deserve future dedicated studies (on other plant tissues and according to a time-basis). In fact, Ca^2+^, BR, and ET concentration changes are also known to modulate plant growth and to proceed with different rates (Hepler, 2005; Yu et al., 2018), and variable levels of these molecules have been reported in other tissues after fungal challenge (Navazio et al., 2007).

This study also demonstrated a clear activation (23 over-represented proteins) of a number of protein machineries involved in cellular vesicle trafficking in fruits from treated plants (Supplementary Figure S8); these vesicles have been reported as essential structures to dump out high- and low-mass compounds involved in plant innate immunity. In fact, since antimicrobials are also toxic to plant cells themselves, they have to be safely delivered to target sites in a separate compartment (Yun and Kwon, 2017). Because immune responses generally require energy otherwise used for the other metabolic processes, it is also very important to properly control duration/strength of these secretory activities. This can be achieved by regulating the sensing of immune signals and the delivery/discharge of extracellular immune molecules, all of which are controlled by membrane trafficking in plant cells. Thus, components of the clathrin coated vesicle machinery, coat protein I and II coatomer machineries, or regulating membrane tethering and fusion were over-represented in fruits from *Trichoderma*-treated plants, suggesting also in this case the occurrence of this physiological phenomenon (Supplementary Figure S8). Among above-mentioned immunity-related components, worth mentioning are exocyst complexes that were already reported to transport anthocyanins between different plant cell subcellular compartments (Pecenková et al., 2017). Preliminary evidence of quantitative changes in few of the above-mentioned protein families were observed in leaves of grapevine plants challenged with *T. harzianum* T39 (Perazzolli et al., 2016).

Finally, a number of proteins (52 in number) known to elicit a protective action against biotic/abiotic stresses showed variable quantitative levels in fruits from fungal-treated plants, highlighting a significant remodeling of the corresponding defense effector molecular machineries (Supplementary Figures S4, S7). Significant differences were observed among either the effect of different *Trichoderma* strains (with most frequent changes in GV41) and components belonging to the same protein family. Regarding heat shock proteins (HSPs), chaperones and protein disulfide-isomerases (PDIs), *T. virens* GV41 was observed to increase the representation of different HSP70 isoforms, HSP91 and DNAJ protein, while it decreased the concentration of various chaperones and HSP20; depending on the isoform, PDIs showed variable levels (Supplementary Figure S4). No significant changes were observed in the case of *T. harzianum* T22 and TH1. On the other hand, a number of proteins involved in the plant response to abiotic stresses also showed quantitative variations. This is the case of some CBS domain-containing protein and late embryogenesis abundant protein isoforms, as well as temperature-induced lipocalin, stress-inducible protein, stress induced protein, universal stress protein A, stress response protein, mucin 22-like protein and metallothionein, which were generally down-represented in *T. virens* GV41 and over-represented in *T. harzianum* T22 (Supplementary Figures S4, S7). A similar quantitative trend was also observed for proteins involved in the plant response to biotic stresses, namely cysteine and serine protease inhibitors, thaumatin domain-containing protein, MLP-like protein 28, Bet v I type allergen, Fra a 1-E allergen chain A and Fra A3 allergen chain B, which generally showed reduced levels in *T. virens* GV41 and a higher representation in *T. harzianum* T22 and TH1. Data on antioxidant proteins have already been described in previous paragraphs. Various defense components reported above have already been demonstrated to show quantitative variations in root and leaf tissues of bean, maize, tomato, cucumber, and grapevine plants treated with *Trichoderma* strains (Marra et al., 2006; Segarra et al., 2007; Shoresh and Harman, 2008; Perazzolli et al., 2016; Manganiello et al., 2018; Nogueira-Lopez et al., 2018; De Palma et al., 2019). The distinct pattern of the allergens present in strawberry fruits depending on the *Trichoderma* spp. used for plant treatment prompts us to suggest the development of future investigations with the aim of evaluating novel food products with specific characteristics for human consumption.

## Conclusion

This study demonstrated that the treatment of strawberry plants with different *Trichoderma* can either influence the relative yield, growth and productivity, as well as the accumulation of anthocyanins and other antioxidants in the corresponding fruits. These findings indicated that the positive effects observed by the application of *Trichoderma*-based products to the developing plant are also transferred to fruits, thus modulating different physiological processes and describing the molecular mechanisms that positively influence food quality and consumer health.

In recent years, with the changing perception by governing bodies to implement precautionary measures that reduce the use of chemical phytosanitary products in agriculture, a great effort has been spent in the development of alternative methods for crop protection. In addition to the valuable influence that *Trichoderma* treatments have demonstrated on the plant, increased strawberry fruit production and enhanced nutritional properties, plus its known success as a biological control agent and its acceptance as a natural product for use in diverse crop production systems, confirms the important role that this beneficial microbe can play in sustainable agricultural while safeguarding the well-being of the consumer and the environment.

## Data Availability Statement

All datasets generated for this study are included in the article/Supplementary Material.

## Author Contributions

NL and SW planned and designed the experiments. NL performed the field research, sample collection, and biometric analysis. AMS, SC, and AS performed the proteomics analysis and interpretation of the data. SW, AS, NL, RM, and FV assisted in the interpretation of results and in the writing of the manuscript. AT and PV defined the experimental protocols for the antioxidant capacity, total phenolic, ascorbic acid, and anthocyanins content in fruits, and performed data elaboration and analyses. All authors contributed to the article and approved the submitted version.

## Conflict of Interest

The authors declare that the research was conducted in the absence of any commercial or financial relationships that could be construed as a potential conflict of interest.

## Footnotes

1 www.fao.org

2 www.arabidopsis.org

3 http://mapman.gabipd.org/web/guest/app/mercator

4 http://string-db.org

## Abbreviations

ASA/DHA: ascorbic acid/dehydroascorbic acid ratio
BCAs: biological control agents
DRPs: differentially represented proteins
GSH/GSSG: reduced glutathione/oxidized glutathione ratio
MRM: multiple reaction monitoring
MS: mass spectrometry
MS/MS: tandem mass spectrometry
TMT: tandem mass tagging.
