# Supplementary figures and images for: Trichoderma Applications on Strawberry Plants Modulate the Physiological Processes Positively Affecting Fruit Production and Quality
Source: Front Microbiol. 2020 Jul 3;11:1364. doi: 10.3389/fmicb.2020.01364 (PMC7350708; doi:10.3389/fmicb.2020.01364)

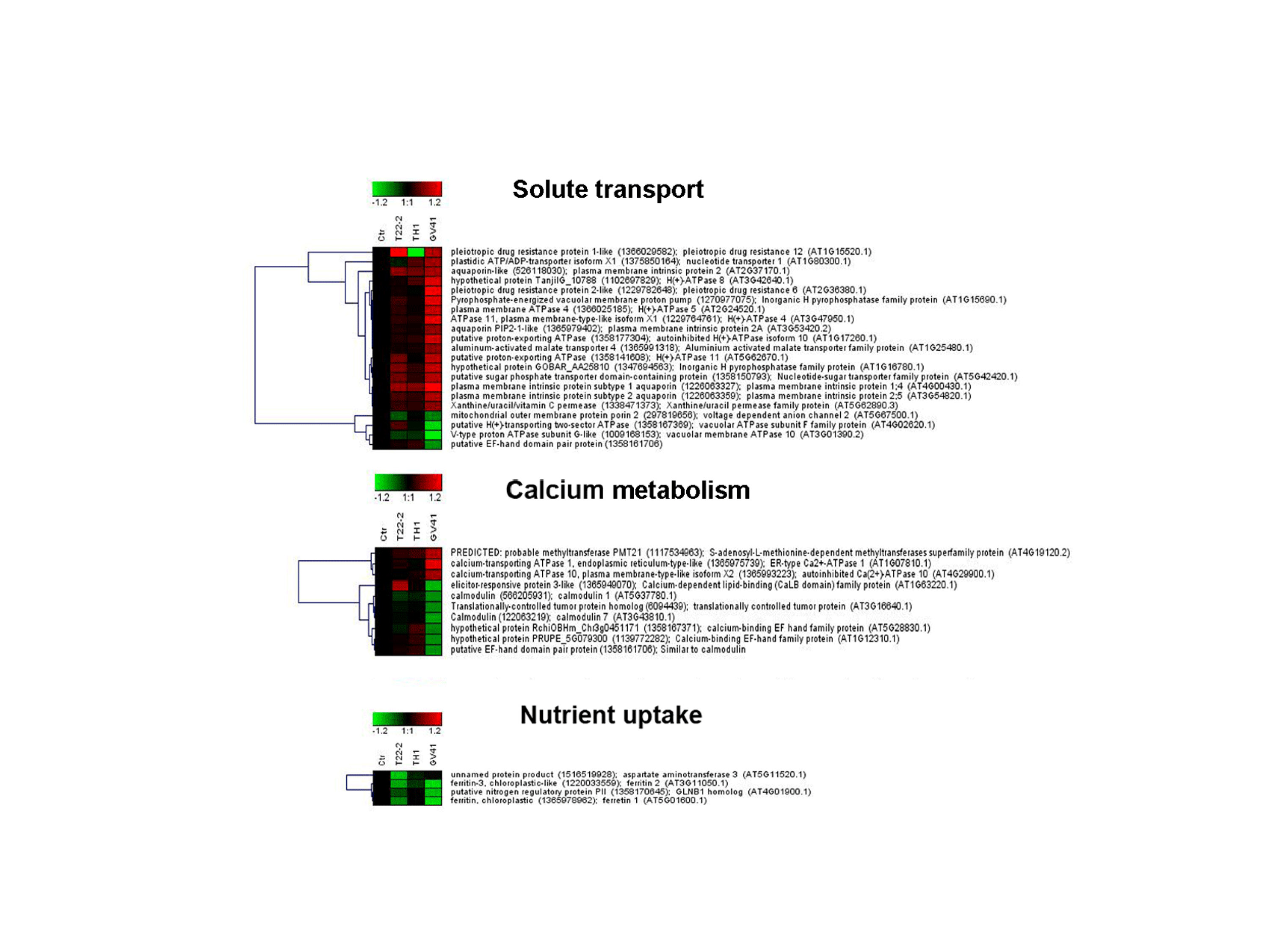

Supplement: Supplementary file 2 [file Image_2.JPEG]

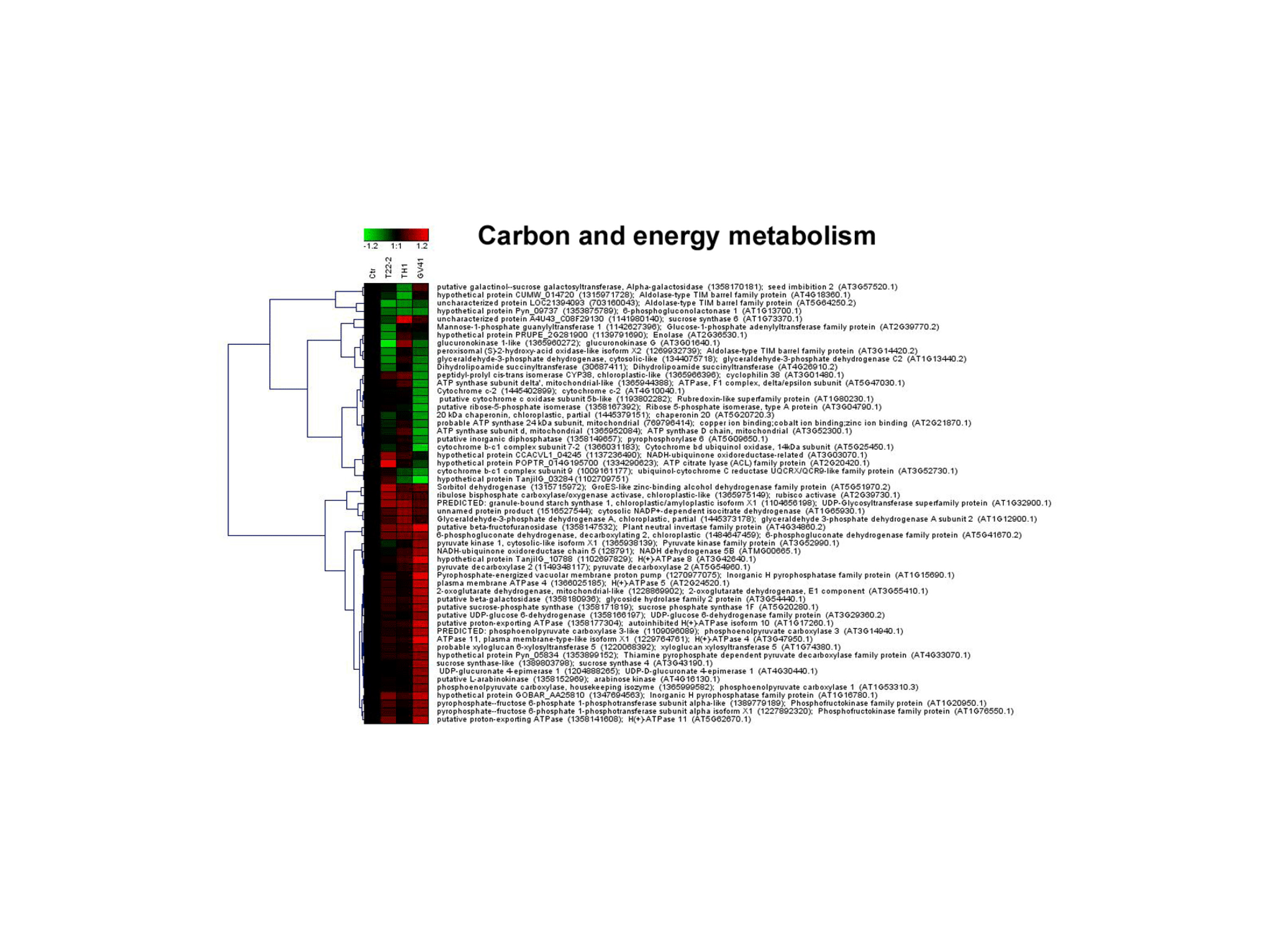

Supplement: Supplementary file 3 [file Image_3.JPEG]

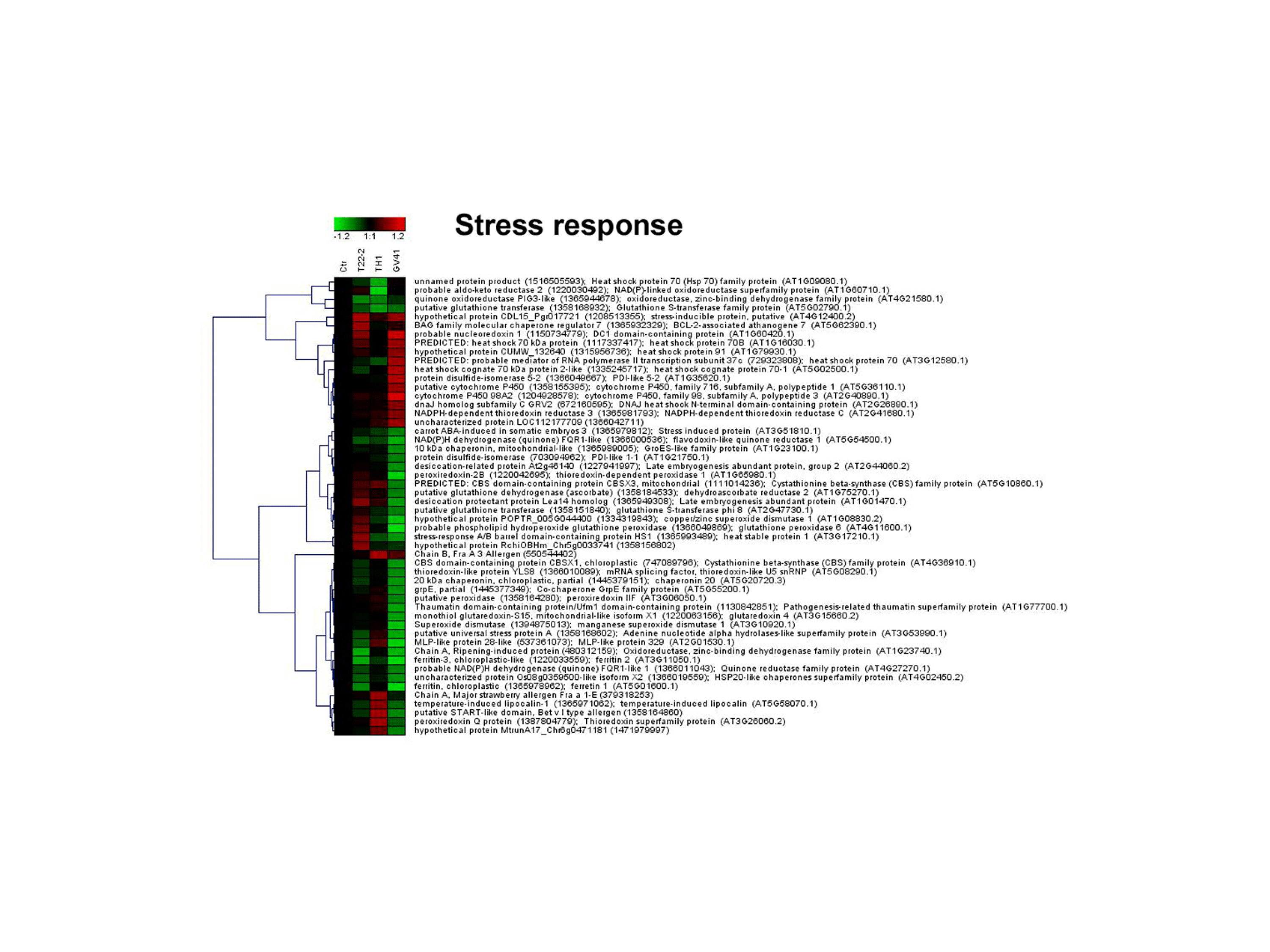

Supplement: Supplementary file 4 [file Image_4.JPEG]

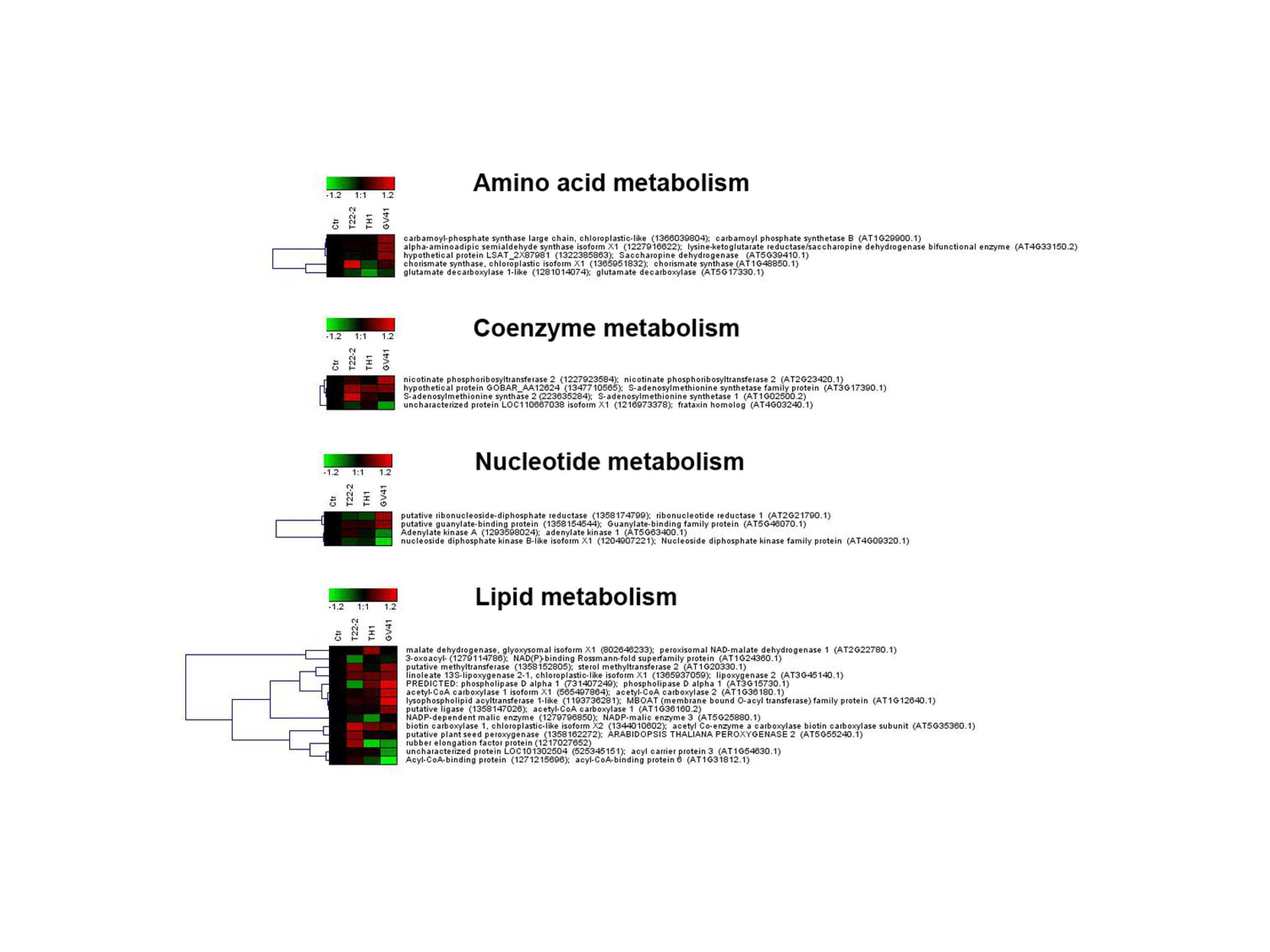

Supplement: Supplementary file 5 [file Image_5.JPEG]

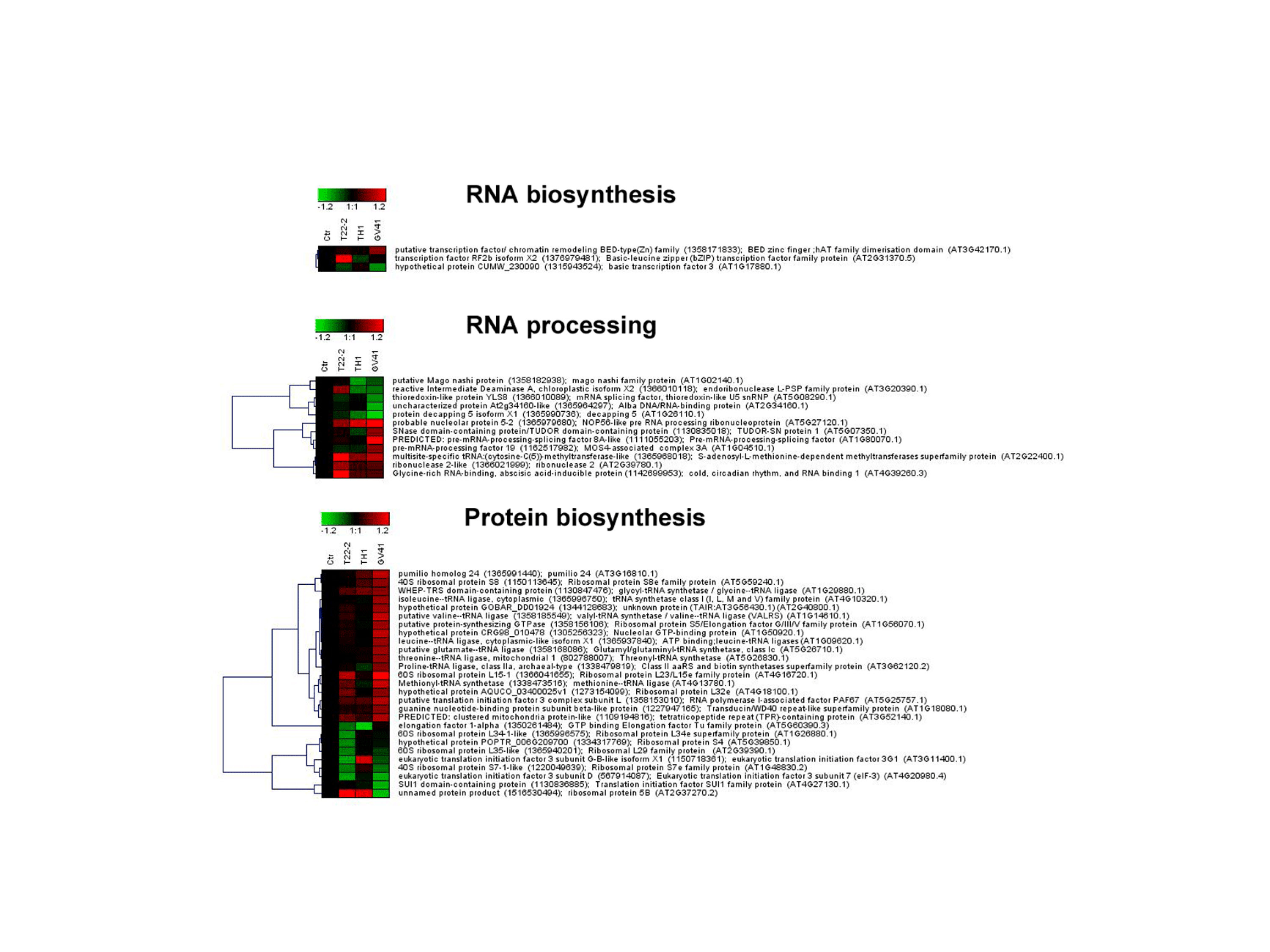

Supplement: Supplementary file 6 [file Image_6.JPEG]

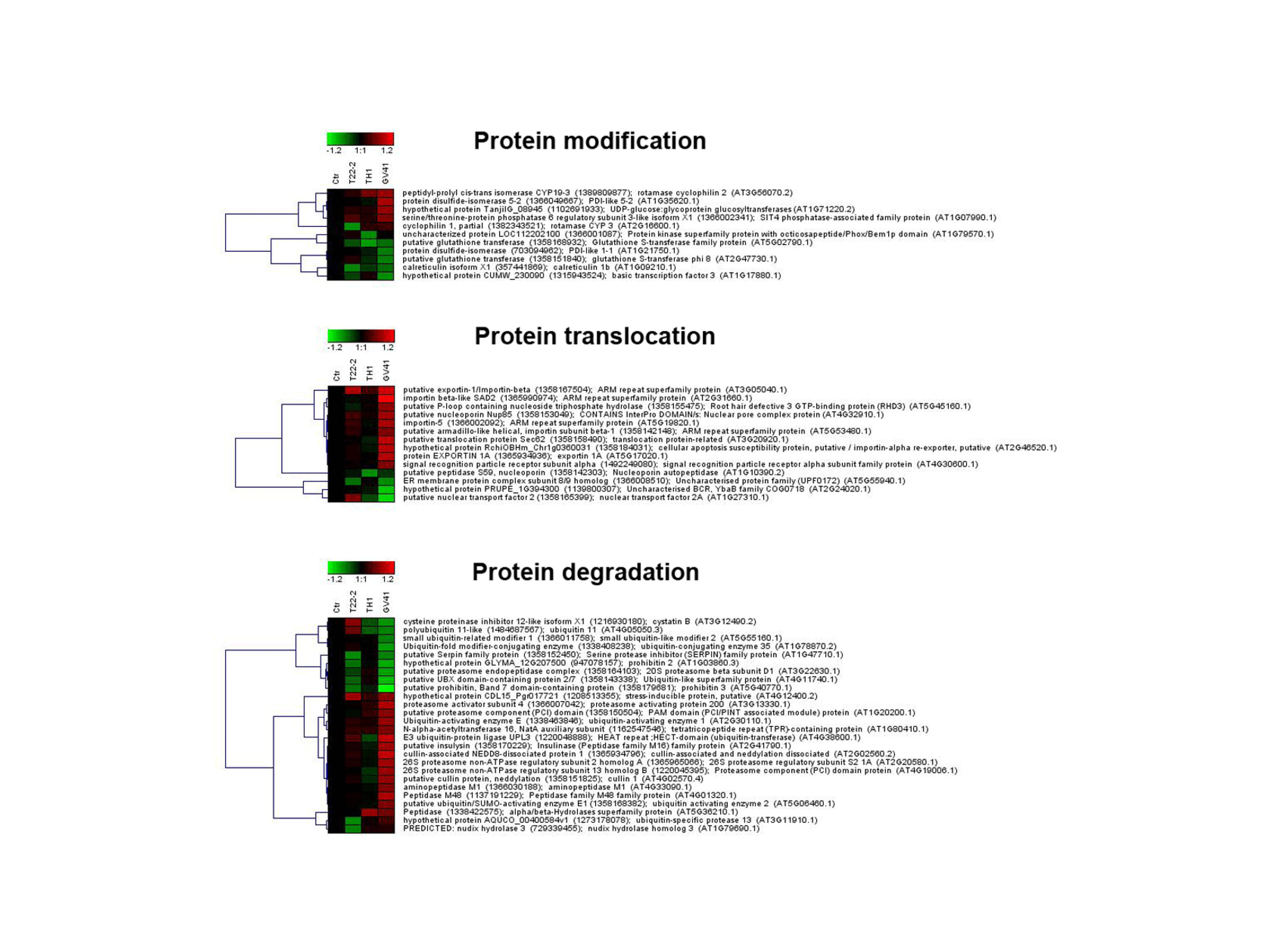

Supplement: Supplementary file 7 [file Image_7.JPEG]

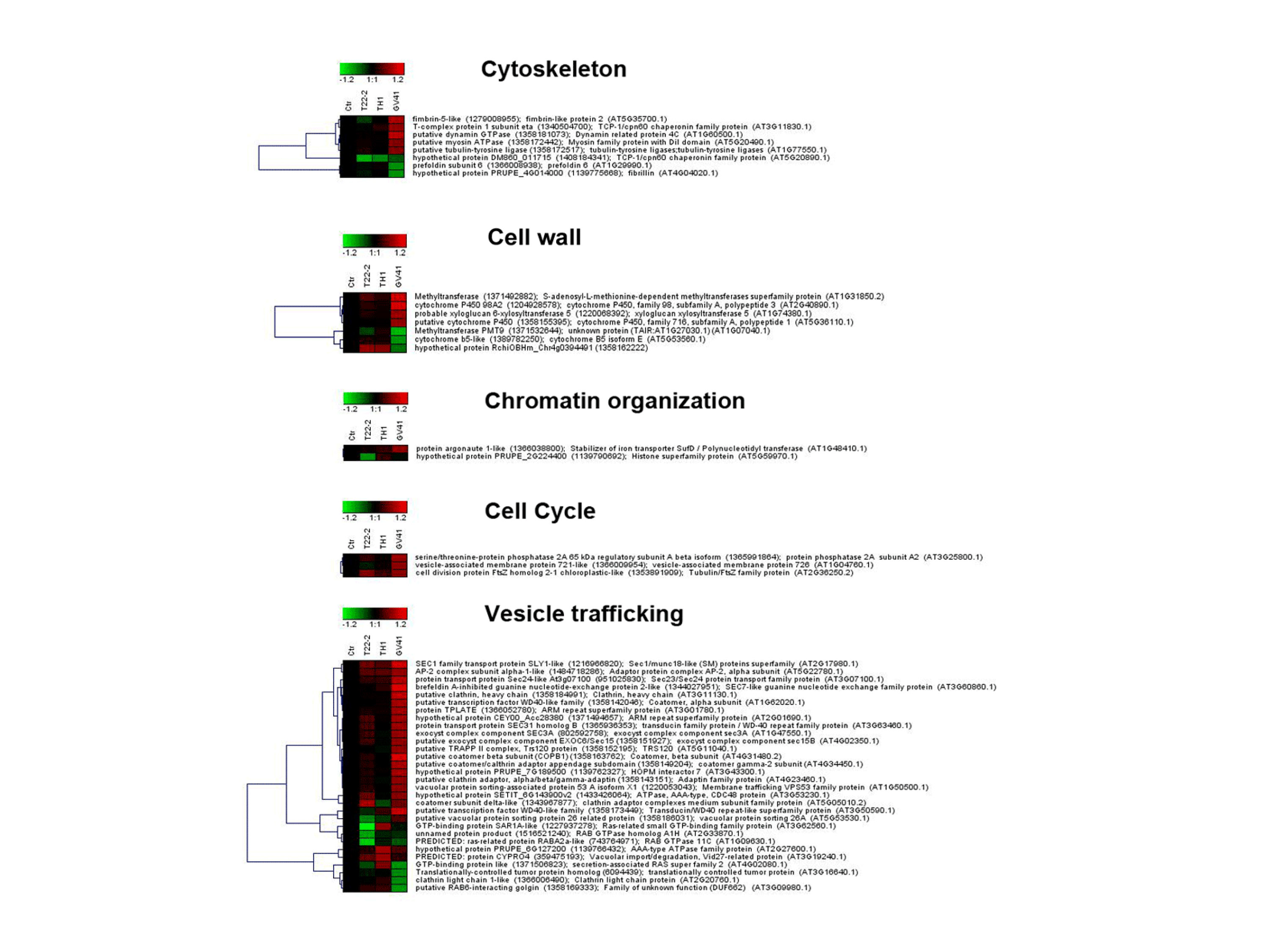

Supplement: Supplementary file 8 [file Image_8.JPEG]

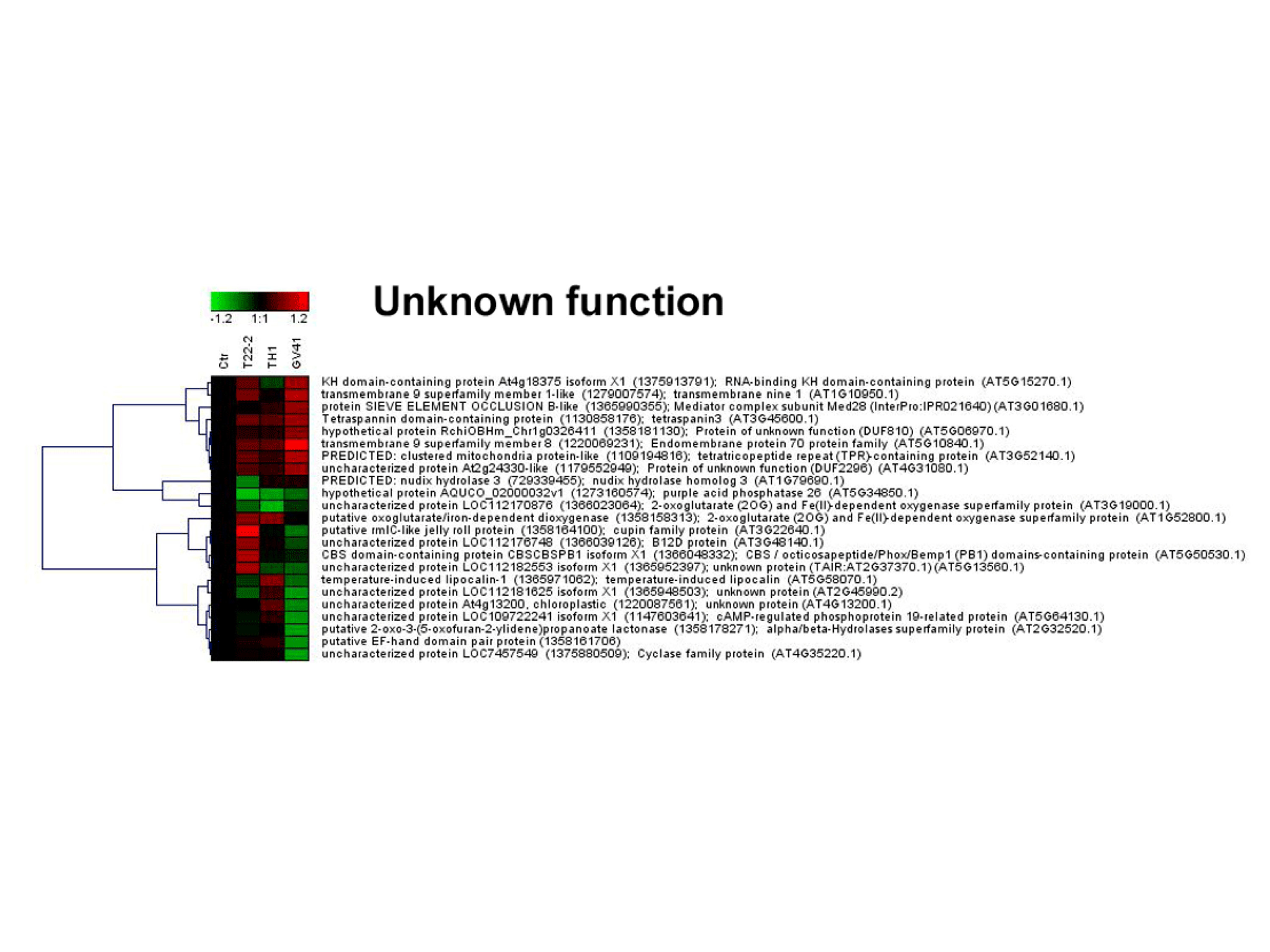

Supplement: Supplementary file 9 [file Image_9.JPEG]
