# Supplementary material for: Trichoderma Applications on Strawberry Plants Modulate the Physiological Processes Positively Affecting Fruit Production and Quality
Source: Front Microbiol. 2020 Jul 3;11:1364. doi: 10.3389/fmicb.2020.01364 (PMC7350708; doi:10.3389/fmicb.2020.01364)
Supplement: Supplementary file 10 [file Data_Sheet_1.zip › Presentation 1.pdf]

SUPPLEMENTARY MATERIAL

Supplementary Table S1. Anthocyanins determined in strawberry samples by LC-DAD-ESI-MS/MS analysis, including peak number, retention time (RT), precursor and product ions, their tentative identification and abbreviation.

Peak	RT (min)	Precursor ion $[M\text{-}H]^+$	Product ion	Tentative identification	Abbreviation
**1**	11.2	449	287	cyanidin 3-*O*-glucoside	cya 3-*O*-glc
**2**	12.8	433	271	pelargonidin 3-*O*-glucoside	pel 3-*O*-glc
**3**	13.5	579	271	pelargonidin 3-*O*-rutinoside	pel 3-*O*-rut
**4**	14.5	519	271	pelargonidin 3-*O*-malonyl-glucoside	pel 3-*O*-mal-glc
**5**	15.1	475	271	pelargonidin 3-*O*-acetyl-glucoside	pel 3-*O*-ac-glc
**6**	17.0	449	287	cyanidin derivative	cya der

Supplementary Table S2. Identification and quantification details of the proteins quantified in this study. Protein accession and description, exp. *q*-value, sum posterior error probability (PEP) score, sequence coverage (%), number of identified peptides, peptide spectrum matches (PSMs), protein groups, number of identified unique peptides, number of razor peptides and Mascot identification score are reported, together with protein theoretical values (number of amino acids, molecular mass, pI). Specific information on protein quantification are reported including: Found in file, Found in sample, modification(s), abundance ratios, abundance (grouped), abundance (scaled), abundance, abundance (normalized). TAIR accession number for homologues counterparts in *Arabidopsis thaliana* and results of Blast alignments are reported for the identified proteins.

Supplementary Table S3. Protein quantitative changes ascertained in fruits from plants treated with different *Trichoderma* strains, with respect to control. Functional assignment of proteins was performed as reported in the experimental section. Figures containing resulting outputs are also reported.

Supplementary Table S4. Top-15 entries deriving from functional enrichment analysis of strawberry DRPs after *Trichoderma* strain treatments. Results from Biological process (GO), Molecular function (GO) and KEGG pathways enrichment are shown.

**Biological Process (GO)**			
GO-term	***Description***	***Count in gene set***	***False-discovery rate***
GO:0010035	response to inorganic substance	59 of 795	2.33e-25
GO:0010038	response to metal ion	44 of 414	2.98e-24
GO:0046686	response to cadmium ion	38 of 286	5.61e-24
GO:0042221	response to chemicals	90 of 2654	1.82e-17
GO:0050896	response to stimulus	124 of 5064	3.16e-14
GO:0051179	localization	75 of 2244	8.12e-14
GO:0006810	transport	71 of 2140	7.59e-13
GO:0044281	small molecule metabolic process	58 of 1503	7.90e-13
GO:0055086	nucleobase-containing small molecule metabolic process	29 of 414	1.31e-11
GO:0009117	nucleotide metabolic process	26 of 323	1.31e-11
GO:0055114	oxidation-reduction process	52 of 1348	1.62e-11
GO:0006950	response to stress	81 of 2932	4.76e-11
GO:0019752	carboxylic acid metabolic process	40 of 863	5.70e-11
GO:0033036	macromolecule localization	38 of 818	2.00e-10
GO:0019637	organophosphate metabolic process	31 of 547	2.01e-10

**Molecular Function (GO)**			
GO-term	***Description***	***Count in gene set***	***False-discovery rate***
GO:0043167	ion binding	120 of 5070	2.55e-12
GO:0005488	binding	170 of 8611	2.55e-12
GO:0003824	catalytic activity	148 of 7239	1.82e-11
GO:0036094	small molecule binding	76 of 2633	4.31e-11
GO:0043168	anion binding	74 of 2629	2.55e-10
GO:0000166	nucleotide binding	70 of 2461	6.15e-10
GO:0016491	oxidoreductase activity	45 of 1201	1.19e-09
GO:0032553	ribonucleotide binding	64 of 2204	1.74e-09
GO:0043169	cation binding	75 of 2949	8.74e-09

GO:0046872	metal ion binding	74 of 2940	1.74e-08
GO:0032555	purine ribonucleotide binding	60 of 2179	4.23e-08
GO:0035639	purine ribonucleoside triphosphate binding	59 of 2147	5.59e-08
GO:0005507	copper ion binding	15 of 157	6.34e-08
GO:0016874	ligase activity	16 of 185	6.54e-08
GO:0019829	cation-transporting ATPase activity	10 of 74	1.50e-06

KEGG Pathways

Pathway	*Description*	*Count in gene set*	*False-discovery rate*
ath01100	Metabolic pathways	70 of 1899	2.51e-15
ath01110	Biosynthesis of secondary metabolites	44 of 1063	5.48e-11
ath00190	Oxidative phosphorylation	17 of 149	3.40e-10
ath01200	Carbon metabolism	19 of 261	1.99e-08
ath04141	Protein processing in endoplasmic reticulum	15 of 205	8.89e-07
ath00970	Aminoacyl-tRNA biosynthesis	8 of 57	1.20e-05
ath00710	Carbon fixation in photosynthetic organisms	8 of 69	3.80e-05
ath04144	Endocytosis	10 of 142	0.00013
ath00620	Pyruvate metabolism	8 of 85	0.00013
ath03013	RNA transport	9 of 161	0.0015
ath00480	Glutathione metabolism	7 of 98	0.0017
ath00010	Glycolysis / Gluconeogenesis	7 of 115	0.0039
ath03050	Proteasome	5 of 58	0.0052
ath00640	Propanoate metabolism	4 of 33	0.0052
ath00030	Pentose phosphate pathway	5 of 58	0.0052

Supplementary Table S5. Bridged and non-linked nodes identified during STRING analysis of DRPs present in strawberry fruits after plant treatment with *Trichoderma* strains (T22, TH1 and GV41). Functional protein associations were based on data recorded for *A. thaliana* protein homologues, whose name is also provided in the table.

**TAIR entry**	**Symbol**	**Description**
AT1G01470	LEA14	Probable desiccation-related protein LEA14;
AT1G01800	AT1G01800	Short-chain dehydrogenase/reductase 2b-like;
AT1G02140	MAGO	Protein mago nashi homolog;
AT1G02500	SAM1	S-adenosylmethionine synthetase 1;
AT1G03860	PHB2	Prohibitin-2, mitochondrial;
AT1G04510	MAC3A	Pre-mRNA-processing factor 19 homolog 1;
AT1G04760	VAMP726	Putative vesicle-associated membrane protein 726;
AT1G07040	AT1G07040	Methyltransferase PMT9;
AT1G07810	ECA1	Calcium-transporting ATPase 1, endoplasmic reticulum-type;
AT1G07990	AT1G07990	SIT4 phosphatase-associated family protein;
AT1G08420	BSL2	Serine/threonine-protein phosphatase BSL2;
AT1G08830	CSD1	Cytosolic copper/zinc superoxide dismutase;
AT1G09080	BIP3	Probable mediator of RNA polymerase II transcription subunit 37b;
AT1G09210	CRT1b	Calreticulin 1b;
AT1G09620	AT1G09620	Leucine-tRNA ligase, cytoplasmic;
AT1G09630	RAB11c	Ras-related protein RABA2a;
AT1G10390	AT1G10390	Nuclear pore complex protein NUP98A;
AT1G10950	TMN1	Transmembrane 9 superfamily member 1;
AT1G12310	AT1G12310	Calcium-binding EF-hand family protein;
AT1G12640	LPLAT1	MBOAT (membrane bound O-acyl transferase) family protein;
AT1G12900	GAPA-2	Glyceraldehyde-3-phosphate dehydrogenase GAPA2, chloroplastic;
AT1G13440	GAPC2	Glyceraldehyde-3-phosphate dehydrogenase GAPC2, cytosolic;
AT1G13700	PGL1	Probable 6-phosphogluconolactonase 1;
AT1G14610	TWN2	Valyl-tRNA synthetase/valine-tRNA ligase (VALRS);
AT1G15520	ABCG40	Pleiotropic drug resistance protein 1-like;
AT1G15690	AVP1	Pyrophosphate-energized vacuolar membrane proton pump 1;
AT1G16030	Hsp70b	Heat shock 70 kDa protein 5;
AT1G16780	VHP2;2	Pyrophosphate-energized membrane proton pump 3;
AT1G17260	AHA10	Autoinhibited H(+)-ATPase isoform 10;
AT1G17880	BTF3	Basic transcription factor 3 (BTF3);
AT1G18080	ATARCA	Transducin/WD40 repeat-like superfamily protein;
AT1G20200	EMB2719	26S Proteasome non-ATPase regulatory subunit 3 homolog A;
AT1G20330	SMT2	24-Methylenesterol C-methyltransferase 2;
AT1G20950	AT1G20950	Pyrophosphate--fructose 6-phosphate 1-phosphotransferase subunit alpha 1;
AT1G21750	PDIL1-1	Protein disulfide isomerase-like (PDIL) protein;
AT1G23100	AT1G23100	Putative 10kDa chaperonin (CPN10) protein;
AT1G23740	AOR	NADPH-dependent alkenal/one oxidoreductase, chloroplastic;
AT1G24360	AT1G24360	*3-O*xoacyl-[acyl-carrier-protein] reductase, chloroplastic;
AT1G25480	AT1G25480	Aluminium activated malate transporter family protein;
AT1G26110	DCP5	Protein decapping 5;
AT1G26880	AT1G26880	Ribosomal protein L34e superfamily protein;
AT1G27310	NTF2A	Nuclear transport factor 2A;
AT1G29880	AT1G29880	Glycyl-tRNA synthetase/glycine-tRNA ligase;
AT1G29900	CARB	Carbamoyl-phosphate synthase large chain, chloroplastic;
AT1G29990	PFD6	Prefoldin 6;
AT1G31812	ACBP6	Acyl-CoA-binding domain-containing protein 6;
AT1G31850	AT1G31850	S-adenosyl-L-methionine-dependent methyltransferase superfamily protein;
AT1G32900	GBSS1	Granule-bound starch synthase 1, chloroplastic/amyloplastic;
AT1G35620	PDIL5-2	Protein disulfide-isomerase 5-2;
AT1G36160	ACC1	Acetyl-CoA carboxylase 1;
AT1G36180	ACC2	Acetyl-CoA carboxylase 2;
AT1G47550	SEC3A	Exocyst complex component SEC3A;
AT1G47710	SERPIN1	Serine protease inhibitor (SERPIN) family protein;
AT1G48410	AGO1	Stabilizer of iron transporter SufD/Polynucleotidyl transferase;

AT1G48830	AT1G48830	Ribosomal protein S7e family protein;
AT1G48850	EMB1144	Chorismate synthase;
AT1G50500	HIT1	Vacuolar protein sorting-associated protein 53 A;
AT1G50920	AT1G50920	Nucleolar GTP-binding protein 1;
AT1G52800	AT1G52800	Putative oxoglutarate/iron-dependent dioxygenase;
AT1G53310	PPC1	Phosphoenolpyruvate carboxylase 1;
AT1G54630	ACP3	Acyl carrier protein
AT1G56070	LOS1	Ribosomal protein S5/Elongation factor G/III/V family protein;
AT1G59960	AT1G59960	Non-functional NADPH-dependent codeinone reductase 2;
AT1G60420	AT1G60420	Nucleoredoxin 1;
AT1G60500	DRP4C	Dynamin related protein 4C;
AT1G60690	AT1G60690	Putative perakine reductase;
AT1G60710	ATB2	Probable aldo-keto reductase 2;
AT1G62020	AT1G62020	Coatomer subunit alpha-1;
AT1G63220	AT1G63220	Elicitor-responsive protein 3-like protein;
AT1G65930	cICDH	Cytosolic NADP+-dependent isocitrate dehydrogenase;
AT1G65980	TPX1	Peroxiredoxin-2B;
AT1G66950	ABCG39	Pleiotropic drug resistance protein 2-like protein;
AT1G71220	EBS1	UDP-glucose:glycoprotein glucosyltransferase;
AT1G73370	SUS6	Sucrose synthase 6;
AT1G74380	XXT5	Probable xyloglucan 6-xylosyltransferase 5;
AT1G74920	ALDH10A8	Betaine aldehyde dehydrogenase
AT1G75270	DHAR2	Glutathione S-transferase DHAR2;
AT1G75280	AT1G75280	NmrA-like negative transcriptional regulator family protein;
AT1G76550	AT1G76550	Pyrophosphate--fructose 6-phosphate 1-phosphotransferase subunit alpha 2;
AT1G77550	AT1G77550	Tubulin-tyrosine ligase;
AT1G77700	AT1G77700	Pathogenesis-related thaumatin superfamily protein;
AT1G78870	UBC35	Ubiquitin-conjugating enzyme E2 35;
AT1G78950	BAS	Beta-amyrin synthase-like protein;
AT1G79570	AT1G79570	Protein kinase with octicosapeptide/Phox/Bem1p domain;
AT1G79690	NUDT3	Nudix hydrolase homolog 3;
AT1G79930	HSP91	Heat shock 70 kDa protein 14;
AT1G80070	EMB14	Pre-mRNA-processing-splicing factor 8A;
AT1G80230	AT1G80230	Cytochrome c oxidase subunit 5b-2, mitochondrial;
AT1G80300	NTT1	ADP/ATP carrier protein 1, chloroplastic;
AT1G80410	EMB2753	N-terminal acetyltransferase A complex auxiliary subunit NAA15;
AT1G80490	T21F11.18	Topless-related protein 1;
AT2G01530	MLP329	MLP-like protein 329 (MLP329);
AT2G01690	AT2G01690	ARM repeat superfamily protein;
AT2G02560	CAND1	Homolog of cullin-associated and neddylation-dissociated protein;
AT2G15220	AT2G15220	Plant basic secretory protein (BSP) family protein;
AT2G16600	ROC3	Peptidyl-prolyl cis-trans isomerase CYP19-1;
AT2G17980	ATSLY1	Sec1/munc18-like (SM) proteins superfamily;
AT2G20420	AT2G20420	Succinate-CoA ligase [ADP-forming] subunit beta, mitochondrial;
AT2G20580	RPN1A 26S	Proteasome non-ATPase regulatory subunit 2 homolog A;
AT2G20760	AT2G20760	Clathrin light chain protein;
AT2G21790	RNR1	Ribonucleoside-diphosphate reductase large subunit;
AT2G21870	MGP1	ATP synthase 24 kDa subunit;
AT2G22400	AT2G22400	Multisite-specific tRNA:(cytosine-C(5))-methyltransferase-like;
AT2G22780	PMDH1	Peroxisomal NAD-malate dehydrogenase 1;
AT2G23420	NAPRT2	Nicotinate phosphoribosyltransferase 2;
AT2G24020	AT2G24020	Nucleoid-associated protein At2g24020, chloroplastic;
AT2G24520	HA5	ATPase 5, plasma membrane-type;
AT2G24940	MAPR2	Steroid-binding protein 3;
AT2G26890	GRV2	DNAJ heat shock N-terminal domain-containing protein;
AT2G27600	SKD1	Protein suppressor of K(+) transport growth defect 1;
AT2G30110	UBA1	Ubiquitin-activating enzyme (E1);
AT2G31370	AT2G31370	Transcription factor RF2b;
AT2G31660	SAD2	Importin beta-like SAD2;
AT2G32520	AT2G32520	2-Oxo-3-(5-oxofuran-2-ylidene)propanoate lactonase;
AT2G33870	ArRABA1h	Ras-related protein RABA1h;
AT2G34160	AT2G34160	Uncharacterized protein At2g34160;

AT2G36250	FTSZ2-1	Cell division protein FtsZ homolog 2-1, chloroplastic;
AT2G36380	ABCG34	Pleiotropic drug resistance protein 2-like;
AT2G36530	LOS2	Bifunctional enolase 2/transcriptional activator;
AT2G37170	PIP2B	Plasma membrane intrinsic protein 2;
AT2G37270	RPS5B	Ribosomal protein S;
AT2G39390	AT2G39390	60S Ribosomal L29 family protein;
AT2G39730	RCA	Ribulose bisphosphate carboxylase/oxygenase activase, chloroplastic;
AT2G39770	CYT1	Mannose-1-phosphate guanylyltransferase 1;
AT2G39780	RNS2	Ribonuclease 2;
AT2G40800	AT2G40800	Import inner membrane translocase subunit;
AT2G40890	CYP98A3	Cytochrome P450, family 98, subfamily A, polypeptide 3;
AT2G41680	NTRC	NADPH-dependent thioredoxin reductase 3;
AT2G41790	AT2G41790	Insulinase (Peptidase family M16) family protein;
AT2G44060	AT2G44060	Late embryogenesis abundant protein, group 2;
AT2G45990	AT2G45990	Ribosomal RNA small subunit methyltransferase G;
AT2G46520	AT2G46520	Cellular apoptosis susceptibility protein, putative/importin-alpha re-exporter;
AT2G47730	GSTF8	Glutathione S-transferase F8, chloroplastic;
AT3G01390	VMA10	V-type proton ATPase subunit G1;
AT3G01480	CYP38	Peptidyl-prolyl cis-trans isomerase CYP38,
AT3G01640	GLCAK	Glucuronokinase 1;
AT3G01680	SEOR1	Protein sieve element occlusion B;
AT3G01780	TPLATE	ARM repeat superfamily protein;
AT3G03070	AT3G03070	NADH dehydrogenase [ubiquinone] iron-sulfur protein 6, mitochondrial;
AT3G04790	EMB3119	Probable ribose-5-phosphate isomerase 3, chloroplastic;
AT3G05040	HASTY	Exportin-1/importin-beta;
AT3G06050	PRXIIF	Peroxiredoxin-2F, mitochondrial;
AT3G07100	ERMO2	Protein transport protein Sec24-like At3g07100;
AT3G09980	AT3G09980	RAB6-interacting golgin;
AT3G10920	MSD1	Superoxide dismutase [Mn] 1, mitochondrial;
AT3G11050	FER2	Ferritin-2, chloroplastic;
AT3G11130	AT3G11130	Clathrin heavy chain 1;
AT3G11400	EIF3G1	Eukaryotic translation initiation factor 3 subunit G;
AT3G11830	AT3G11830	T-complex protein 1 subunit eta;
AT3G11910	UBP13	Ubiquitin carboxyl-terminal hydrolase 13;
AT3G12490	CYSB	Cysteine proteinase inhibitor 6;
AT3G12580	HSP70	Mediator of RNA polymerase II transcription subunit 37c;
AT3G13330	PA200	Proteasome activating protein 200;
AT3G14420	GOX1	Peroxisomal (S)-2-hydroxy-acid oxidase-like;
AT3G14940	PPC3	Cytosolic phosphoenolpyruvate carboxylase;
AT3G15660	GRX4	Monothiol glutaredoxin-S15, mitochondrial;
AT3G15730	PLDALPHA1	Phospholipase D alpha 1;
AT3G15880	WSIP2	Topless-related protein 4;
AT3G16640	TCTP	Translationally controlled tumor protein;
AT3G16810	PUM24	Pumilio homolog 24;
AT3G17210	HS1	Stress-response A/B barrel domain-containing protein HS1;
AT3G17390	MTO3	S-adenosylmethionine synthetase family protein;
AT3G19000	AT3G19000	2-Oxoglutarate (2OG) and Fe(II)-dependent oxygenase superfamily protein;
AT3G19240	AT3G19240	Vacuolar import/degradation, Vid27-related protein;
AT3G20390	AT3G20390	Reactive intermediate deaminase A, chloroplastic;
AT3G20920	AT3G20920	Translocation protein Sec62;
AT3G21790	AT3G21790	UDP-glucose flavonoid *3-O*-glucosyltransferase 3;
AT3G22630	PBD1	Proteasome subunit beta type-2-A;
AT3G22640	PAP85	Vicilin-like seed storage protein At3g22640;
AT3G25800	PP2AA2	Serine/threonine-protein phosphatase 2A 65 kDa regulatory subunit A β isoform;
AT3G26060	PRXQ	Peroxiredoxin Q protein;
AT3G29360	UGD2	UDP-glucose 6-dehydrogenase family protein;
AT3G42170	DAYSLEEPER	Zinc finger BED domain-containing protein DAYSLEEPER;
AT3G42640	HA8	ATPase 8, plasma membrane-type;
AT3G43190	SUS4	Sucrose synthase 4;
AT3G43300	ATMIN7	Brefeldin A-inhibited guanine nucleotide-exchange protein 5;
AT3G43810	CAM7	Calmodulin-7;
AT3G45140	LOX2	Linoleate 13S-lipoxygenase 2-1;

AT3G45600	TET3	Tetraspanin-3;
AT3G47950	HA4	ATPase 4, plasma membrane-type;
AT3G48140	AT3G48140	B12D protein;
AT3G48890	MAPR3	Putative progesterone-binding protein homolog (Atmp2) mRNA;
AT3G50590	AT3G50590	Transducin/WD40 repeat-like superfamily protein;
AT3G51730	AT3G51730	Saposin B domain-containing protein;
AT3G51810	EM1	Stress induced protein;
AT3G52140	NOXY38	Tetratricopeptide repeat (TPR)-containing protein;
AT3G52300	ATPQ	ATP synthase subunit d, mitochondrial;
AT3G52730	AT3G52730	Cytochrome b-c1 complex subunit 9;
AT3G52990	AT3G52990	Pyruvate kinase 1;
AT3G53230	AtCDC48B	Cell division control protein 48 homolog D;
AT3G53260	PAL2	Phenylalanine ammonia-lyase 2;
AT3G53420	PIP2A	Plasma membrane intrinsic protein subfamily PIP2;
AT3G53990	AT3G53990	Universal stress protein A;
AT3G54440	AT3G54440	Beta-galactosidase;
AT3G54820	PIP2;5	Plasma membrane intrinsic protein 2;5;
AT3G55410	AT3G55410	2-Oxoglutarate dehydrogenase, E1 component;
AT3G56070	ROC2	Peptidyl-prolyl cis-trans isomerase CYP19-3;
AT3G57520	SIP2	Probable galactinol-sucrose galactosyltransferase 2;
AT3G60860	AT3G60860	Brefeldin A-inhibited guanine nucleotide-exchange protein 2;
AT3G62120	AT3G62120	Proline-tRNA ligase;
AT3G62560	AT3G62560	GTP-binding protein SAR1A-like;
AT3G63460	AT3G63460	Transport protein SEC31 homolog B;
AT4G00430	PIP1;4	Plasma membrane intrinsic protein 1;4;
AT4G01320	ATSTE24	Peptidase family M48 family protein;
AT4G01900	GLB1	Nitrogen regulatory protein P-II homolog;
AT4G02080	SAR2	Secretion-associated RAS super family 2;
AT4G02350	SEC15B	Exocyst complex component EXOC6/SEC15B;
AT4G02450	AT4G02450	HSP20-like chaperones superfamily protein;
AT4G02570	CUL1	Cullin-1;
AT4G02620	AT4G02620	H(+)-transporting two-sector ATPase;
AT4G03240	FH	Frataxin, mitochondrial;
AT4G04020	FIB	Probable plastid-lipid-associated protein 1, chloroplastic;
AT4G05050	UBQ11	Polyubiquitin 11;
AT4G09320	NDPK1	Nucleoside diphosphate kinase B-like isoform;
AT4G10040	CYTC-2	Cytochrome c-2;
AT4G10320	AT4G10320	Isoleucine-tRNA ligase;
AT4G11600	GPX6	Probable phospholipid hydroperoxide glutathione peroxidase 6, mitochondrial;
AT4G11740	SAY1	Plant UBX domain-containing protein 8;
AT4G12400	Hop3	Stress-inducible protein, putative;
AT4G13200	AT4G13200	Uncharacterized protein At4g13200, chloroplastic;
AT4G13780	AT4G13780	Methionine-tRNA ligase, putative/methionyl-tRNA synthetase,
AT4G16130	ARA1	Arabinose kinase;
AT4G16720	AT4G16720	60S ribosomal protein L23/L15e family protein;
AT4G18100	AT4G18100	Ribosomal protein L32e;
AT4G18360	GOX3	Peroxisomal (S)-2-hydroxy-acid oxidase GLO5;
AT4G19006	AT4G19006	26S proteasome non-ATPase regulatory subunit 13 homolog B;
AT4G19120	ERD3	Methyltransferase PMT21;
AT4G20980	AT4G20980	Eukaryotic translation initiation factor 3 subunit 7 (eIF-3);
AT4G21580	AT4G21580	Quinone oxidoreductase PIG3-like;
AT4G23460	AT4G23460	Beta-adaptin-like protein C;
AT4G26910	AT4G26910	Dihydrolipoamide succinyltransferase;
AT4G27130	AT4G27130	Translation initiation factor SUI1 family protein;
AT4G27270	AT4G27270	NAD(P)H dehydrogenase (quinone) FQR1-like 1;
AT4G29900	ACA10	Calcium-transporting ATPase 10, plasma membrane-type;
AT4G30440	GAE1	UDP-D-glucuronate 4-epimerase 1;
AT4G30600	AT4G30600	Signal recognition particle receptor alpha subunit family protein;
AT4G31080	AT4G31080	Integral membrane metal-binding family protein (DUF2296);
AT4G31480	AT4G31480	Coatomer subunit beta-1 (COPB1);
AT4G32910	AT4G32910	Nuclear pore complex protein NUP85;
AT4G33070	AT4G33070	Thiamine pyrophosphate dependent pyruvate decarboxylase family protein;

AT4G33090	APM1	Aminopeptidase M1;
AT4G33150	AT4G33150	Alpha-aminoadipic semialdehyde synthase;
AT4G33640	AT4G33640	Costars family protein At4g33640;
AT4G34450	AT4G34450	Coatomer gamma-2 subunit, putative/gamma-2 coat protein;
AT4G34640	SQS1	Squalene synthase;
AT4G34860	A/N-InvB	Beta-fructofuranosidase;
AT4G35220	AT4G35220	Cyclase family protein;
AT4G36910	LEJ2	CBS domain-containing protein CBSX1, chloroplastic;
AT4G37980	ELI3-1	Cinnamyl alcohol dehydrogenase 7/mannitol dehydrogenase;
AT4G37990	ELI3-2	Cinnamyl alcohol dehydrogenase 8/mannitol dehydrogenase;
AT4G38600	KAK	E3 ubiquitin-protein ligase
AT4G39230	AT4G39230	NmrA-like negative transcriptional regulator family protein;
AT4G39260	GRP8	Glycine-rich RNA-binding, abscisic acid-inducible protein;
AT5G01600	FER1	Ferritin-1, chloroplastic;
AT5G02500	HSC70-1	Heat shock cognate 70 kDa protein 2-like;
AT5G02790	GSTL3	Glutathione S-transferase family protein;
AT5G05010	AT5G05010	Coatomer subunit delta-like;
AT5G06460	UBA2	Ubiquitin/SUMO-activating enzyme E1 2;
AT5G06970	AT5G06970	Protein of unknown function (DUF810);
AT5G07350	Tudor1	Ribonuclease TUDOR 1;
AT5G08290	YLS8	mRNA splicing factor, thioredoxin-like U5 snRNP;
AT5G09650	PPa6	Soluble inorganic pyrophosphatase 6, chloroplastic;
AT5G10840	EMP1	Transmembrane 9 superfamily member 8;
AT5G10860	CBSX3	CBS domain-containing protein CBSX3, mitochondrial;
AT5G11040	TRS120	TRAPP II complex, Trs120 protein;
AT5G11520	ASP3	Aspartate aminotransferase 3, chloroplastic;
AT5G13560	AT5G13560	Structural maintenance of chromosomes protein;
AT5G13930	TT4	Chalcone and stilbene synthase family protein;
AT5G15270	AT5G15270	RNA-binding KH domain-containing protein;
AT5G17020	XPO1A	Protein exportin 1A;
AT5G17330	GAD	Glutamate decarboxylase 1;
AT5G19820	emb2734	Importin 5/uncharacterized protein At5g19820;
AT5G20280	SPS1F	Sucrose phosphate synthase 1F;
AT5G20490	XIK	Myosin family protein with Dil domain having ATPase activity;
AT5G20720	CPN20	20 kDa Chaperonin, chloroplastic;
AT5G20890	AT5G20890	TCP-1/cpn60 chaperonin family protein;
AT5G22780	AT5G22780	Adaptor protein complex AP-2, alpha subunit;
AT5G25450	AT5G25450	Cytochrome bd ubiquinol oxidase, 14kDa subunit;
AT5G25757	AT5G25757.1	Eukaryotic translation initiation factor 3 subunit L;
AT5G25880	NADP-ME3	NADP-dependent malic enzyme (EC 1.1.1.40);
AT5G26710	AT5G26710	Glutamyl/glutaminyl-tRNA synthetase, class Ic;
AT5G26830	AT5G26830	Threonine-tRNA ligase, mitochondrial 1;
AT5G27030	F2P16.14	Topless-related protein 3;
AT5G27120	AT5G27120	NOP56-like pre RNA processing ribonucleoprotein;
AT5G28830	AT5G28830	Calcium-binding EF hand family protein;
AT5G34850	PAP26	Bifunctional purple acid phosphatase 26;
AT5G35160	AT5G35160	Endomembrane protein 70 protein family;
AT5G35360	CAC2	Acetyl Co-enzyme a carboxylase biotin carboxylase subunit;
AT5G35700	FIM5	Fimbrin-like protein 2;
AT5G36110	CYP716A1	Cytochrome P450, family 716, subfamily A, polypeptide 1;
AT5G36210	AT5G36210	Peptidase belonging to the alpha/beta-Hydrolases superfamily;
AT5G37780	CAM1	Calmodulin involved in thigmomorphogenesis;
AT5G39410	AT5G39410	Mitochondrial saccharopine dehydrogenase-like oxidoreductase At5g39410;
AT5G39850	AT5G39850	Ribosomal protein S4;
AT5G40770	PHB3	Prohibitin-3, mitochondrial;
AT5G41670	AT5G41670	6-Phosphogluconate dehydrogenase, decarboxylating 2, chloroplastic;
AT5G42420	AT5G42420	Nucleotide-sugar phosphate transporter family protein;
AT5G45160	RL2	Root hair defective 3 GTP-binding protein;
AT5G46070	AT5G46070	Guanylate-binding family protein;
AT5G47030	AT5G47030	ATP synthase subunit delta', mitochondrial;
AT5G50530	CBSCBSPB4	CBS/octicosapeptide/Phox/Bemp1 domains-containing protein CBSCBSPB1;
AT5G51970	AT5G51970	Sorbitol dehydrogenase;

AT5G53480	AT5G53480	Armadillo-like helical, importin subunit beta-1;
AT5G53530	VPS26A	Vacuolar protein sorting-associated protein 26A;
AT5G53560	CB5-E	Cytochrome b5 isoform E;
AT5G54500	FQR1	NAD(P)H dehydrogenase (quinone) FQR1-like protein;
AT5G54960	PDC2	Pyruvate decarboxylase-2; belongs to the TPP enzyme family
AT5G55160	SUMO2	Small ubiquitin-like modifier (SUMO) polypeptide;
AT5G55200	MGE1	GrpE protein homolog 1, mitochondrial;
AT5G55240	ATPXG2	Arabidopsis thaliana peroxigenase 2;
AT5G55940	emb2731	ER membrane protein complex subunit 8/9 homolog;
AT5G58070	TIL	Temperature-induced lipocalin-1;
AT5G59240	AT5G59240	40S ribosomal protein S8;
AT5G59970	At1g07660	Histone superfamily protein;
AT5G60390	At1g07940	Elongation factor 1-alpha;
AT5G62390	BAG7	BAG family molecular chaperone regulator 7;
AT5G62670	HA11	ATPase 11, plasma membrane-type;
AT5G62890	AT5G62890	Xanthine/uracil/vitamin C permease family protein;
AT5G63400	At5g63400	Adenylate kinase 4;
AT5G64130	AT5G64130	cAMP-regulated phosphoprotein 19-related protein;
AT5G64250	AT5G64250	2-Nitropropane dioxygenase-like protein;;
AT5G67500	VDAC2	Mitochondrial outer membrane protein porin 2;
ATMG0066	NAD5B	NADH-ubiquinone oxidoreductase chain 5;
AT2G47780	REF	Rubber elongation factor protein.

Figure legends

Supplementary Figure S1. HPLC-DAD chromatogram of an exemplificative strawberry dried extract recorded at 520 nm. Putative compound identification was performed according to the scientific literature (Holzwarth 2012; Carbone et al., 2009). (1) cyanidin 3-*O*-glucoside; (2) pelargonidin 3-*O*-glucoside; (3) pelargonidin 3-*O*-rutinoside; (4) pelargonidin 3-*O*-malonyl-glucoside; (5) pelargonidin 3-*O*-acetyl-glucoside; (6) cyanidin derivative.

Supplementary Figure S2. Heat-map representation and hierarchical clustering analysis of proteins involved in solute transport (upper panel), calcium metabolism (middle panel) and nutrient uptake (lower panel), which were differentially represented in strawberry fruits produced by plants subjected to the treatments with *Trichoderma* strains (T22, TH1 and GV41), as compared to control (Ctr). Shown are proteins showing abundance fold changes ≥1.50 or ≤0.66 with respect to control ($P \leq 0.05$) (Supplementary Table S3). Data are reported as log_2 transformed abundance ratio values.

Supplementary Figure S3. Heat-map representation and hierarchical clustering analysis of proteins involved in carbon and energy metabolism that were differentially represented in strawberry fruits produced by plants subjected to the treatments with *Trichoderma* strains (T22, TH1 and GV41), as compared to control (Ctr). Shown are proteins showing abundance fold changes ≥1.50 or ≤0.66 with respect to control ($P \leq 0.05$) (Supplementary Table S3). Data are reported as log_2 transformed abundance ratio values.

Supplementary Figure S4. Heat-map representation and hierarchical clustering analysis of proteins involved in stress response that were differentially represented in strawberry fruits produced by plants subjected to the treatments with *Trichoderma* strains (T22, TH1 and GV41), as compared to control (Ctr). Shown are proteins showing abundance fold changes ≥1.50 or ≤0.66 with respect to control ($P \leq 0.05$) (Supplementary Table S3). Data are reported as log_2 transformed abundance ratio values.

Supplementary Figure S5. Heat-map representation and hierarchical clustering analysis of proteins involved in amino acid metabolism (upper panel), coenzyme metabolism (middle panel), nucleotide metabolism (middle panel) or lipid metabolism (lower panel), which were differentially represented in strawberry fruits produced by plants subjected to the treatments with *Trichoderma*

strains (T22, TH1 and GV41), as compared to control (Ctr). Shown are proteins showing abundance fold changes ≥1.50 or ≤0.66 with respect to control (P≤ 0.05) (Supplementary Table S3). Data are reported as log_2 transformed abundance ratio values.

Supplementary Figure S6. Heat-map representation and hierarchical clustering analysis of proteins involved in RNA biosynthesis (upper panel), RNA processing (middle panel) and protein biosynthesis (lower panel), which were differentially represented in strawberry fruits produced by plants subjected to the treatments with *Trichoderma* strains (T22, TH1 and GV41), as compared to control (Ctr). Shown are proteins showing abundance fold changes ≥1.50 or ≤0.66 with respect to control (P≤ 0.05) (Supplementary Table S3). Data are reported as log_2 transformed abundance ratio values.

Supplementary Figure S7. Heat-map representation and hierarchical clustering analysis of proteins involved in protein modification (upper panel), protein translocation (middle panel) and protein degradation (lower panel), which were differentially represented in strawberry fruits produced by plants subjected to the treatments with *Trichoderma* strains (T22, TH1 and GV41), as compared to control (Ctr). Shown are proteins showing abundance fold changes ≥1.50 or ≤0.66 with respect to control (P≤ 0.05) (Supplementary Table S3). Data are reported as log_2 transformed abundance ratio values.

Supplementary Figure S8. Heat-map representation and hierarchical clustering analysis of proteins involved in cytoskeleton (upper panel), cell wall (middle upper panel), chromatin organization (middle panel), cell cycle (middle lower panel) and vesicle trafficking (lower panel), which were differentially represented in strawberry fruits produced by plants subjected to the treatments with *Trichoderma* strains (T22, TH1 and GV41), as compared to control (Ctr). Shown are proteins showing abundance fold changes ≥1.50 or ≤0.66 with respect to control (P≤ 0.05) (Supplementary Table S3). Data are reported as log_2 transformed abundance ratio values.

Supplementary Figure S9. Heat-map representation of hierarchical clustering analysis of proteins with unknown function that were differentially represented in strawberry fruits produced by plants subjected to the treatments with *Trichoderma* strains (T22, TH1 and GV41), as compared to control (Ctr). Shown are proteins showing abundance fold changes ≥1.50 or ≤0.66 with respect to control (P≤ 0.05) (Supplementary Table S3). Data are reported as log_2 transformed abundance ratio values. Results related to proteins with unknown function are shown.

References

Holzwarth, M., Korhummel, S., Carle, R., and Kammerer, D. R. (2012). Evaluation of the Effects of Different Freezing and Thawing Methods on Color, Polyphenol and Ascorbic Acid Retention in Strawberries (*Fragaria* × *Ananassa* Duch.). *Food Res. Int.* 48, 241–248. doi: 10.1016/j.foodres.2012.04.004

Carbone, F., Preuss, A., De Vos, R. C. H., D'Amico, E., Perrotta, G., Bovy, A. G., et al. (2009). Developmental, Genetic and Environmental Factors Affect the Expression of Flavonoid Genes, Enzymes and Metabolites in Strawberry Fruits. *Plant, Cell Environ*. doi: 10.1111/j.1365-3040.2009.01994
